# Operational greenhouse-gas emissions of deep learning in digital pathology: a modelling study

**DOI:** 10.1016/S2589-7500(23)00219-4

**Published:** 2023-11-22

**Authors:** Alireza Vafaei Sadr, Roman Bülow, Saskia von Stillfried, Nikolas E J Schmitz, Pourya Pilva, David L Hölscher, Peiman Pilehchi Ha, Marcel Schweiker, Peter Boor

**Affiliations:** a Institute of Pathology, University Hospital Aachen, Rheinisch-Westfälische Technische Hochschule Aachen, Aachen, Germany; b Healthy Living Spaces Lab, Institute for Occupational, Social and Environmental Medicine, Medical Faculty, Rheinisch-Westfälische Technische Hochschule Aachen, Aachen, Germany; c Department of Nephrology and Immunology, Rheinisch-Westfälische Technische Hochschule Aachen, Aachen, Germany; d Department of Public Health Sciences, College of Medicine, Pennsylvania State University, Hershey, PA, USA

## Abstract

**Background:**

Deep learning is a promising way to improve health care. Image-processing medical disciplines, such as pathology, are expected to be transformed by deep learning. The first clinically applicable deep-learning diagnostic support tools are already available in cancer pathology, and their number is increasing. However, data on the environmental sustainability of these tools are scarce. We aimed to conduct an environmental-sustainability analysis of a theoretical implementation of deep learning in patient-care pathology.

**Methods:**

For this modelling study, we first assembled and calculated relevant data and parameters of a digital-pathology workflow. Data were breast and prostate specimens from the university clinic at the Institute of Pathology of the Rheinisch-Westfälische Technische Hochschule Aachen (Aachen, Germany), for which commercially available deep learning was already available. Only specimens collected between Jan 1 and Dec 31, 2019 were used, to omit potential biases due to the COVID-19 pandemic. Our final selection was based on 2 representative weeks outside holidays, covering different types of specimens. To calculate carbon dioxide (CO_2_) or CO_2_ equivalent (CO_2_ eq) emissions of deep learning in pathology, we gathered relevant data for exact numbers and sizes of whole-slide images (WSIs), which were generated by scanning histopathology samples of prostate and breast specimens. We also evaluated different data input scenarios (including all slide tiles, only tiles containing tissue, or only tiles containing regions of interest). To convert estimated energy consumption from kWh to CO_2_ eq, we used the internet protocol address of the computational server and the Electricity Maps database to obtain information on the sources of the local electricity grid (ie, renewable *vs* non-renewable), and estimated the number of trees and proportion of the local and world's forests needed to sequester the CO_2_ eq emissions. We calculated the computational requirements and CO_2_ eq emissions of 30 deep-learning models that varied in task and size. The first scenario represented the use of one commercially available deep-learning model for one task in one case (1-task), the second scenario considered two deep-learning models for two tasks per case (2-task), the third scenario represented a future, potentially automated workflow that could handle 7 tasks per case (7-task), and the fourth scenario represented the use of a single potential, large, computer-vision model that could conduct multiple tasks (multitask). We also compared the performance (ie, accuracy) and CO_2_ eq emissions of different deep-learning models for the classification of renal cell carcinoma on WSIs, also from Rheinisch-Westfälische Technische Hochschule Aachen. We also tested other approaches to reducing CO_2_ eq emissions, including model pruning and an alternative method for histopathology analysis (pathomics).

**Findings:**

The pathology database contained 35 552 specimens (237 179 slides), 6420 of which were prostate specimens (10 115 slides) and 11 801 of which were breast specimens (19 763 slides). We selected and subsequently digitised 140 slides from eight breast-cancer cases and 223 slides from five prostate-cancer cases. Applying large deep-learning models on all WSI tiles of prostate and breast pathology cases would result in yearly CO_2_ eq emissions of 7·65 metric tons (t; 95% CI 7·62–7·68) with the use of a single deep-learning model per case; yearly CO_2_ eq emissions were up to 100·56 t (100·21–100·99) with the use of seven deep-learning models per case. CO_2_ eq emissions for different deep-learning model scenarios, data inputs, and deep-learning model sizes for all slides varied from 3·61 t (3·59–3·63) to 2795·30 t (1177·51–6482·13. For the estimated number of overall pathology cases worldwide, the yearly CO_2_ eq emissions varied, reaching up to 16 megatons (Mt) of CO_2_ eq, requiring up to 86 590 km^2^ (0·22%) of world forest to sequester the CO_2_ eq emissions. Use of the 7-task scenario and small deep-learning models on slides containing tissue only could substantially reduce CO_2_ eq emissions worldwide by up to 141 times (0·1 Mt, 95% CI 0·1–0·1). Considering the local environment in Aachen, Germany, the maximum CO_2_ eq emission from the use of deep learning in digital pathology only would require 32·8% (95% CI 13·8–76·6) of the local forest to sequester the CO_2_ eq emissions. A single pathomics run on a tissue could provide information that was comparable to or even better than the output of multitask deep-learning models, but with 147 times reduced CO_2_ eq emissions.

**Interpretation:**

Our findings suggest that widespread use of deep learning in pathology might have considerable global-warming potential. The medical community, policy decision makers, and the public should be aware of this potential and encourage the use of CO_2_ eq emissions reduction strategies where possible.

**Funding:**

German Research Foundation, European Research Council, German Federal Ministry of Education and Research, Health, Economic Affairs and Climate Action, and the Innovation Fund of the Federal Joint Committee.

## Introduction

The increase in greenhouse gases (ie, carbon dioxide [CO_2_], methane, nitrous oxide, and fluorinated gases) is considered to be the main driver of global warming and climate change.^1^ To measure and compare the global-warming potential of greenhouse gases, their relative emissions are standardised as CO_2_ equivalents (CO_2_eq) in metric tons. CO_2_eq emissions are increasing due to increasing energy demands, which are partly due to digitisation and computational demands.^2^ Addressing this environmental challenge would align with the UN Sustainable Development Goals.^3^

The digitisation of workflows and processes has happened in many areas of medicine, including pathology. Digital pathology is the scanning of histological glass slides to generate digital whole-slide images (WSIs). This digitisation process enables the effective use of computational techniques and computer vision (ie, the development of algorithms to enable machines to interpret and analyse visual data) for the advanced analysis of histopathological images. WSIs in pathology are among the largest datatypes in clinical medicine.^4^ In our experience, a single pathology case can consist of up to 100 WSIs and a single pathology institute can produce several hundred to thousands of WSIs per day.

Deep learning is one of the most promising computational techniques in pathology^5^, ^6^, ^7^, ^8^, ^9^, ^10^, ^11^, ^12^ as it can undertake numerous tasks (eg, automating quantitative image analysis), making the diagnostic process faster and more efficient than analogue pathology.^13^, ^14^, ^15^ Deep learning can also support diagnostic decision making in disease classification, prognostication, or treatment response and has the potential to reveal novel insights that might not be discernible to pathologists, such as convolutional neural networks that can recognise patterns in images which humans are unable to see.^16^, ^17^ Deep-learning algorithms typically require substantial computational resources. Newly developed deep-learning models are becoming larger than before and thereby computationally more demanding, driving increasing energy consumption.^18^ In medical applications, including pathology, the main—or even only—focus in deep-learning development is on improving performance; energy consumption and global-warming potential are rarely considered.^19^ The failure to recognise energy consumption and global-warming potential could be due to little data on these consequences of deep-learning implementation.

We aimed to conduct an environmental-sustainability analysis of a theoretical implementation of deep learning in patient-care pathology, focusing on operational CO_2_eq emission.

## Methods

### Study design and data

For this modelling study, we first assembled and calculated relevant data and parameters of a digital-pathology workflow. Data were breast and prostate specimens from the University Hospital at the Institute of Pathology of the Rheinisch-Westfälische Technische Hochschule Aachen (Aachen, Germany), for which commercially available deep-learning models were already available. Only specimens collected between Jan 1 and Dec 31, 2019 were used, to omit potential biases due to the COVID-19 pandemic. We queried the pathology database within the Institute of Pathology laboratory information system for all slides, all prostate slides, and all breast slides and selected and subsequently digitised some. Our selection was based on a representative 2 weeks outside holidays, covering different types of specimens.

**Research in context**

**Evidence before this study**

We searched PubMed, Web of Science, Google Scholar, and arXiv using the search terms “Pathology” AND/OR “Artificial Intelligence” AND/OR “Deep Learning” AND/OR “Sustainability” for research published in English between database inception and March 30, 2023. Literature cited in identified articles was also incorporated; additional data from the Our World in Data, Global Burden of Disease, and TechPowerUp databases were checked. To our knowledge, no previous research has investigated the environmental effects of using deep-learning-based systems in pathology.

**Added value of this study**

To our knowledge, this is the first study investigating the global-warming potential of using deep learning in pathology by calculating the greenhouse-gas emissions of various deep-learning-based systems. We provide a point of reference against which comparisons can be made, comparing potential outcomes, that might inform future decision making in the development and implementation of these systems.

**Implications of all the available evidence**

This study emphasises the urgent need to consider sustainability when developing and implementing deep-learning-based systems in digital pathology. Policy makers and researchers should work towards sustainable deep-learning practices.

To calculate CO_2_ equivalent (CO_2_eq) emissions of deep learning in pathology, we gathered relevant data for exact numbers and sizes of WSIs, which were generated by scanning histopathology samples of the two selected use cases, prostate and breast. We considered examples of computational devices in which deep learning could be implemented (ie, central processing unit [CPU] and graphics processing unit [GPU]). We considered several scenarios of deep-learning use in pathology, including current and future application scenarios with a single deep-learning model per case (1-task) to up to seven deep-learning models per case (7-task), as well as a general model that was able to tackle multiple tasks (multitask). We gathered information on local energy sources generating electricity for deep learning from Electricity Maps (Copenhagen, Denmark) based on the internet protocol address of the health-care provider's server, which enabled the calculation of emitted CO_2_eq. We extrapolated these calculations for all organs that undergo digital pathology in the Institute of Pathology and on a national and international scale and estimated future developments. We also estimated the effect of potential solutions for reducing the energy consumption of deep learning in pathology.

The correlation between file size and number of pixels suggested that file size alone was insufficient for the calculation of input data for deep learning, and that the tissue proportion of each WSI was an important factor. For subsequent calculations, we therefore defined three scenarios for data input. First, we used all tiles (ie, a small image extracted from a WSI), including tiles without tissue (hereafter referred to as WSI). Second, we used only tiles containing tissue, which were automatically selected by a tissue-detection deep-learning model we developed for this analysis (hereafter referred to as tissue). Third, we used tiles containing regions of interest (ROIs), such as cancer, which were also automatically detected by a dedicated deep-learning model (hereafter referred to as ROI). The second and third scenarios represent data-reduction approaches. For these approaches, experienced pathologists (RB and SvS) annotated tissue and tumours on WSIs using QuPath version 0.4.2.^20^

To extrapolate our results for Germany and the world, we used our collected data, national society's information, and open databases. No available data on the number of pathology cases and slides exist at the national level in Germany. Therefore, we used the estimation of cancer cases in Germany based on the Global Burden of Diseases, Injuries, and Risk Factors Study (GBD) database and the proportion of cancer versus non-cancer cases in our institute in 2019. This process enabled us to calculate CO_2_eq data that could be compared with other countries and international estimates. No international estimates of the number of pathology cases and slides exist. Therefore, we used a similar approach as for national estimates to calculate the overall number of pathology cases worldwide. In both national and international contexts, we used the count of cancer cases and the ratio of cancer to non-cancer cases to estimate the number of pathology cases for other countries by cross multiplication in the proportion. Subsequently, the estimation of CO_2_eq is also projected contingent upon the renewable energy contribution within each respective country.

The number of model parameters was a more reliable, although not precise, indicator for CO_2_eq calculation than the number of model layers.^21^ Previously, floating point operations per second, multiply-adds, number of trainable parameters, and hardware-time use have been suggested as not strongly correlated with CO_2_eq emissions.^21^ Therefore, we focused on operational emissions, using the experiment-impact-tracker^21^ to measure real-time activities of the hardware device on which a deep-learning model was running. This framework allowed for a precise calculation of the energy consumption associated with the operation of dynamic random-access memory, CPU, GPU, data input, data output, and the internal cooling system of the computer.

To convert estimated energy consumption from kWh to CO_2_eq, we used the internet protocol address of the computational server and the Electricity Maps database to obtain information on the sources of the local electricity grid (ie, renewable *vs* non-renewable). For the calculation of the number of trees needed to sequester CO_2_eq emissions, we used the Arbor Day Foundation report, which states that an average mature tree can absorb about 21·8 kg of CO_2_eq per year.^22^ We acknowledge that this value varies largely depending on the type of tree and other factors (eg, diameter).^23^ To estimate the number of trees needed to compensate a given amount of CO_2_eq, the amount of CO_2_eq is divided by 21·8. To calculate the area of the world's forest required for CO_2_eq sequestration (and the equivalent for local and national forests), we used the estimated overall capacity of the world's forest to sequester CO_2_eq emissions (7·6 Gt)^24^ and the estimated total forest area (40·6 million km^2^) worldwide.

The retrospective example data were used in anonymised fashion in accordance with the declaration of Helsinki and approved by the local ethics review board (EK23-094). The aggregated workflow process data do not require ethics approval (waived by the local ethics review board).

### CO_2_ eq calculations and statistical analysis

We calculated the computational requirements and CO_2_eq emissions of the most common deep-learning models on Keras that were used for histopathology classification (20 classification models) and segmentation (10 segmentation models). We selected deep-learning models from each category (ie, segmentation and classification) representing small, medium, and large deep learning based on minimum, median, and maximum identified CO_2_eq emission data.

We defined four deep-learning implementation scenarios in pathology. The first scenario represented the use of one commercially available deep-learning model for one task in one case, which is current practice, specifically breast and prostate cancer cases (hereafter referred to as 1-task). The second scenario considered two deep-learning models for two tasks per case (hereafter referred to as 2-task)—one model was a classifier (eg, assisting diagnostics and prioritising cases) and the other generated segmentation (eg, assisting in detection, area analysis, and ROI selection for subsequent analysis). The third scenario represented a future, potentially automated workflow that could handle 7 tasks per case (ie, prioritise cases and conduct tissue annotation, ROI annotation, instance annotation, grading, molecular-alteration detection, treatment-response prediction, and survival analysis) via seven deep-learning models (hereafter referred to as 7-task). The fourth scenario represented the use of a single potential, large, computer-vision model that could conduct multiple tasks (hereafter referred to as multitask). This scenario was based on the current success of large language models such as Generative Pre-Trained Transformer (GPT)-4^25^ and Pathways Language Model (PaLM) 540-billion parameter (540B)^26^ in various domains. Such large models have also been described for image-based analysis^27^ and might be useful for multimodal biomedical-data integration, as has been shown for PaLM-540B.^28^

To generate comparable CO_2_eq emissions values for normalised computational requirements, we ran all deep-learning models on the same defined amount of 10^7^ tiles for all models. We customised and updated the experiment-impact-tracker^21^ to reconcile with the available Python version.

On the basis of calculated CO_2_eq emissions for each of these deep-learning models, we selected models with low (minimum identified) CO_2_eq emissions, with medium (median) CO_2_eq emissions, and with high (maximum) CO_2_eq emissions for both segmentation and classification. Because no exact data on CO_2_eq emissions for large, multitask deep-learning models exist, we used PaLM-540B^25^, ^29^ as it is one of the biggest available multitask models and used the CO_2_eq emission mean value for this model for subsequent calculations. To generate this value, we used statistical estimation based on the number of parameters in PaLM and statistical modelling for the number of parameters versus measured CO_2_eq emission for the 30 models. Due to the use of estimation, exact CO_2_eq emission might differ for specific tasks (appendix pp 1–2).

We next considered the influence of computational devices. Deep-learning-based CO_2_eq emissions are dependent on computational devices (ie, the hardware). GPUs are more efficient than CPUs for computing image-based deep-learning operations because of their parallel processing capabilities,^30^ which is particularly relevant for large deep-learning models. Although in our experience more CPUs are available in clinics, for further calculations we used data for Nvidia GPU Quadro 6000 due to its higher efficiency than CPUs and its increased affordability and usability at desktop workstations compared with other Nvidia GPU servers (ie, A100 and V100).

We have evaluated different data-reduction strategies and investigated their effect on CO_2_eq emissions, which showed that simple deep-learning models (defined as having fewer parameters) perform similarly to advanced deep-learning models in various pathology tasks.^5^, ^10^, ^31^ To test this hypothesis in regard to CO_2_eq emissions, we compared the performance (ie, accuracy) and CO_2_eq emissions of 13 different deep-learning models for the classification of renal cell carcinoma (RCC) on WSIs (appendix pp 21–22).^32^ We collected 265 consecutive RCC cases from the archives of the Institute of Pathology (University Hospital, Rheinisch-Westfälische Technische Hochschule Aachen), including cases from Jan 1, 2011, to Dec 31, 2018, and specifically selecting major RCC subtypes (papillary, clear cell, and chromophobe RCCs). Another approach to reducing the computational requirements of deep-learning models is pruning.^33^, ^34^ Pruning in machine learning trims less important components from a model to enhance efficiency and reduce complexity. We assessed classification accuracy before and after pruning. A further potential approach to reduce CO_2_eq emissions could be the use of alternative methods for histopathology analysis (eg, pathomics).^35^ Pathomics uses two sequential deep-learning models for tissue detection and segmentation, then large-scale extraction of quantitative morphological features and biostatistical analysis. We used the CO_2_eq emissions for UNet-2D run-on data to simulate a pathomics workflow. We assumed that all required outcomes could be delivered by the extracted morphological features.

We next estimated the potential development of CO_2_eq emissions in pathology at the national and international levels, considering four main parameters (ie, development of renewable-energy sources, development of deep-learning models, improvement of computational resources, and projections on cancer cases (as a proxy for the expected number of histopathology cases). For each parameter, we used existing data (appendix p 22) and bootstrapping to generate a statistical model for the estimation (appendix pp 3–5).

Analyses were done with Python (version 3.10) on Linux servers (Ubuntu 22.04.1) with specific configurations of a Python environment and the following libraries: cucim (version 22.2.0), cudatoolkit (version 11.6.0), cudnn (version 8.2.1.32), experiment-impact-tracker (version 0.1.9), Keras (version 2.11.0), Keras-unit-collection (version 0.1.13), openslide (version 3.4.1), openslide-python (version 1.1.2), and TensorFlow (version 2.11.0).

### Role of the funding source

The funders of this study had no role in study design, data collection, data analysis, data interpretation, writing of the report, or the decision to submit for publication.

## Results

**Figure 1:**
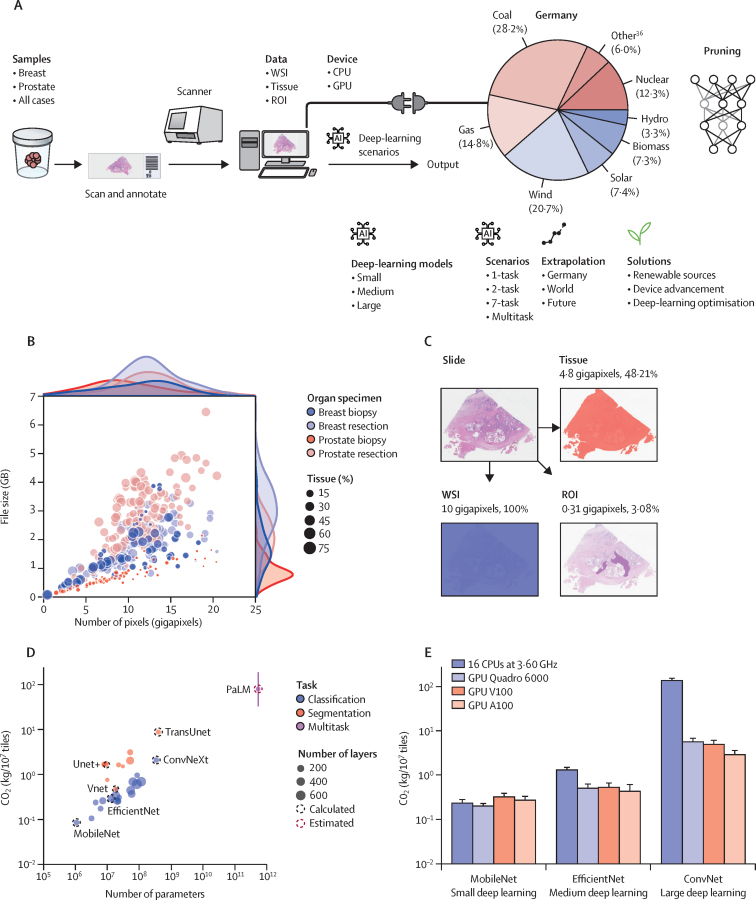
Study workflow and data (A) Study workflow.^36^ (B) Number of WSIs and their slides used in this study. Circles denote one WSI, with the size of the circle corresponding to the percentage of slide that was occupied by tissue. The upper axis shows the overall distribution of the number of pixels and the right axis shows the overall distribution of the number of file sizes. The association between file size and number of pixels is not linear because of the scanner compression algorithm. (C) An example of a prostate-resection WSI stained with haematoxylin and eosin. Three different data input strategies are shown (ie, entire WSI, tissue only, or ROI only; for tissue and ROI, the percentages use WSI as the denominator). (D) CO_2_eq emissions of deep-learning models calculated on the basis of German energy versus the number of deep-learning-model parameters, comparing the 30 deep-learning classification and segmentation models. Each circle denotes one deep learning; circle size is proportional to the number of deep-learning layers. For further calculations, six deep-learning models were selected—three for classification and three for segmentation tasks. To model potential use of a general, multitask model, values for the PaLM model were statistically estimated. (E) CO_2_eq emission for 10^7^ tiles with different computational devices for classification. The GPU Quadro 6000 was chosen for further calculation because of the trade-off between performance and affordability. AI=artificial intelligence. CO_2_=carbon dioxide. CO_2_eq=CO_2_ equivalent. CPU=central processing unit. GB=gigabytes. GPU=graphics processing unit. PaLM=Pathways Language Model. ROI=region of interest. t=tons. WSI=whole-slide image.

The pathology database within the Institute of Pathology laboratory provided 35 552 (15·0%) of 237 179 slides, 6420 (63·5%) of which were prostate specimens (10 115 slides) and 11 801 (59·7%) of which were breast specimens (19 763 slides), collected between Jan 1 and Dec 31, 2019. We selected and subsequently digitised 140 slides from eight breast-cancer cases (ie, two ultrasound-guided needle biopsies; two magnetic resonance tomography-guided vacuum-aspiration biopsies; two lumpectomy specimens; and two mastectomy specimens, including sentinel lymph nodes) and 223 slides from five prostate-cancer cases (ie, two prostate magnetic-resonance–ultrasound fusion biopsies; one transurethral prostate-resection specimen; and two prostatectomy specimens, including lymph nodes and preprostatic fat tissue). WSIs varied in number of pixels and the proportion of tissue per WSI and case (figure 1B; appendix p 6).

We calculated the computational requirements and CO_2_eq emissions of 30 deep-learning models (figure 1D). We chose 10 segmentation and 20 classification deep-learning models—we used all 10 available segmentation models. For classification models, 71 were available in Keras and Keras-unit-collection. From these 71, we selected 20 (28%) of varying depth to cover the range of model complexity and represent the three different model sizes. On the basis of calculated CO_2_eq emissions for each of these 30 models, we selected one model each with low, medium, and high CO_2_eq emissions for both segmentation and classification (ie, six models in total; figure 1D).

Image-compression techniques indicated a moderate correlation (r=0·58) between file size and number of pixels (figure 1B). This correlation suggested that file size alone was insufficient for the calculation of input data for deep learning, and that the tissue proportion of each WSI was an important factor.

**Figure 2:**
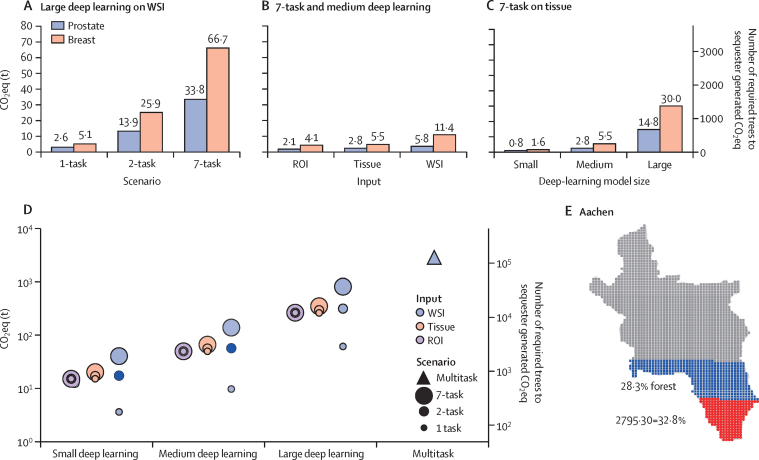
Yearly CO_2_ or CO_2_ eq emissions of the use of deep learning in a single pathology centre (Institute of Pathology, University Hospital Aachen, Rheinisch-Westfälische Technische Hochschule Aachen, Aachen, Germany) (A–C) CO_2_eq emissions variation based on the different variables for prostate (blue) and breast (red) cases. (D) CO_2_eq emissions based on the various parameters extrapolated to all cases that were analysed in the pathology centre. (E) Visualisation of required local forest area to sequester the CO_2_eq emissions of all cases with a multitask model. Map displays Aachen, where the pathology centre is located. Aachen forest area is depicted in blue. Red dots superimposed on blue dots indicate the amount of forest required to sequester produced CO_2_eq. CO_2_=carbon dioxide. CO_2_eq=CO_2_ equivalent. ROI=region of interest. t=tons. WSI=whole-slide image.

Using the acquired parameters, data, and calculations of number of WSI tiles and CO_2_eq emission for the included models, we analysed the yearly CO_2_eq emissions of our Institute of Pathology (Rheinisch-Westfälische Technische Hochschule Aachen, Aachen, Germany; appendix p 11). Applying large deep-learning models on all WSI tiles of prostate and breast pathology cases would result in yearly CO_2_eq emissions of 7·65 tons (t; 95% CI 7·62–7·68) in the 1-task scenario; yearly CO_2_eq emissions were up to 100·56 t (100·21–100·99) in the 7-task scenario (figure 2A). Data-reduction and model-reduction strategies could substantially decrease emitted CO_2_eq (figure 2B, C). In the 7-task scenario, the use of different data inputs (ie, WSI *vs* tissue *vs* ROI) and model sizes (ie, small *vs* medium *vs* large) could reduce CO_2_eq emissions up to 53 times (appendix pp 11–12). We also converted CO_2_eq into number of trees needed to offset and absorb emitted CO_2_eq (figure 2A–C).

CO_2_eq emissions for different implementation scenarios, data inputs, and deep-learning model sizes for all slides varied from 3·61 t (3·59–3·63) to 2795·30 t (1178·51–6482·13; figure 2D; appendix p 12). Considering the local environment in Aachen, Germany, the maximum CO_2_eq emission from the use of deep learning in digital pathology only would require 32·8% (95% CI 13·8–76·6) of the local forest to sequester the CO_2_eq emissions (figure 2E). Currently, the estimated local forest is 28·31% of Aachen.

The use of entire WSIs as data input results in excessive CO_2_eq, whereas use of an ROI can lead to the loss of contextual tissue information. Therefore, we used the tissue-containing tiles only (hereafter referred to as tissue) that were automatically selected by a dedicated deep-learning model for all extrapolations.

**Figure 3:**
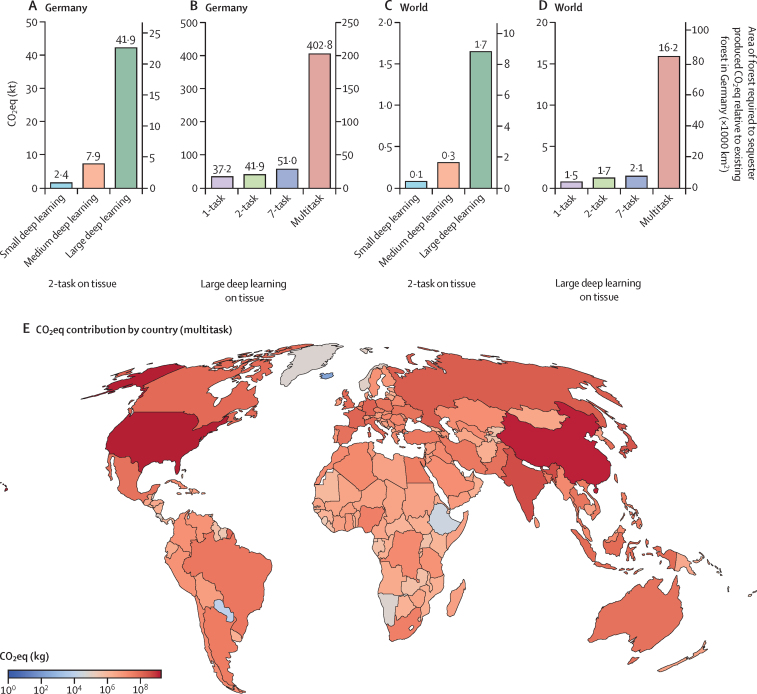
National and global extrapolation of yearly CO_2_ or CO_2_ eq emissions for the use of deep learning in pathology (A, B) Extrapolation of CO_2_eq emission for all pathology cases in Germany. (C, D) Extrapolation of CO_2_eq emission for all pathology cases worldwide. The right axis indicates the required forest area to sequester produced CO_2_eq emissions relative to the whole existing forest in Germany (A, B) or the world (C, D). (E) Estimated CO_2_eq contribution per country if a multitask model is used. CO_2_=carbon dioxide. CO_2_eq=CO_2_ equivalent. kt=kilotons.

The proportion of cancer versus non-cancer cases in our institute was 4804 versus 35 552; alongside the estimation of 552 800 cancer cases in Germany, this resulted in 4 643 837 total cases in Germany in 2019. Based on a survey of the German Association of Pathologists, there are 17 550 000 estimated cases per year in Germany.^37^ Therefore, with our approach, the number of cases might underestimate the absolute CO_2_eq emissions approximately by 3·8 times. The national yearly CO_2_eq emissions varied by up to 806 times due to the sizes of models and scenarios (0·50 kilotons [kt], 0·50–0·50 for 1-task, WSI, small deep learning *vs* 3·61 kt, 3·60–3·63 for 1-task, WSI, small deep learning), which would require up to 2151 km^2^ (95% CI 906–4988; 1·9%) of Germany's forest to sequester the emitted CO_2_eq (figure 3A, B; appendix p 13).

Our estimate of the overall number of pathology cases worldwide was 173 265 243. Yearly CO_2_eq emissions varied based on the various parameters, reaching up to 16 megatons (Mt; 95% CI 6·8–37·6) of CO_2_eq, requiring up to 86 590 km^2^ (36 476–200 796; 0·22%, 0·09–0·51) of the world's forest to sequester the emissions (figure 3C, D). Use of the 7-task scenario and small deep-learning models on tissue could substantially reduce CO_2_eq emission by up to 141 times (0·1 Mt, 0·1–0·1; appendix pp 14–15). Based on the Global Burden of Disease database, emitted CO_2_eq distribution for the multitask scenario might differ between countries (figure 3E; appendix p 16).

**Figure 4:**
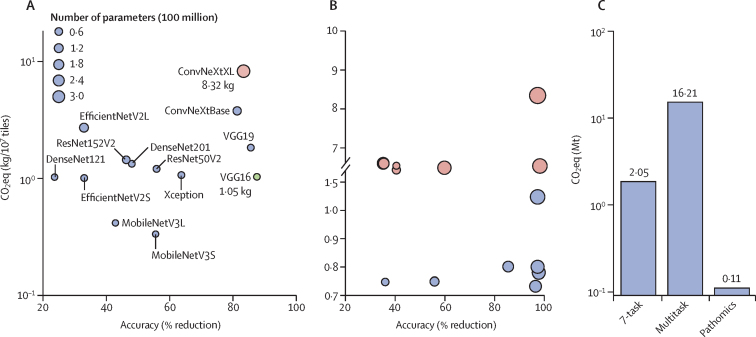
Potential solutions to reduce CO_2_ or CO_2_ eq emissions with a deep-learning model classifying renal cell carcinoma (A) CO_2_eq emissions of different deep-learning models for classification of renal cell carcinoma. The size of each circle corresponds to the number of parameters of the deep-learning model. 13 models were obtained for comparison. Red (ConvNeXtXL) and blue and green (VGG16) models were selected for the pruning experiment. (B) CO_2_eq emissions of the two selected models, pruned up to 70%. The size of each circle corresponds to remnant % of the red (ConvNeXtXL) and blue (VGG16) models that were selected for pruning. Analysis was done for each model, including the unpruned model. (C) Comparison of deep-learning models with an alternative approach for histopathology analysis (ie, pathomics). CO_2_=carbon dioxide. CO_2_eq=CO_2_ equivalent. Mt=megatons.

We compared the performance and CO_2_eq emissions of 13 different deep-learning models for the classification of RCC on WSIs (appendix p 2).^32^ The depth of the model did not reflect its RCC-classification accuracy (figure 4A; appendix p 21). Despite having fewer trainable parameters, VGG16, a relatively simple deep-learning model, outperformed ConvNeXt, a deeper model, while reducing CO_2_eq emissions by approximately 8 times (figure 4A; appendix pp 21–22). Furthermore, when these two RCC-classification models had been pruned by up to 40% (ie, VGG16 pruned 40% and ConvNeXt pruned 20%) they retained their accuracy while generating 20–30% less CO_2_eq emissions than non-pruned models (figure 4B; appendix p 22). A single pathomics run on a tissue could provide information that was comparable to or even better than the output of deep-learning models, but with 147 times reduced CO_2_eq emissions (figure 4C).

**Figure 5:**
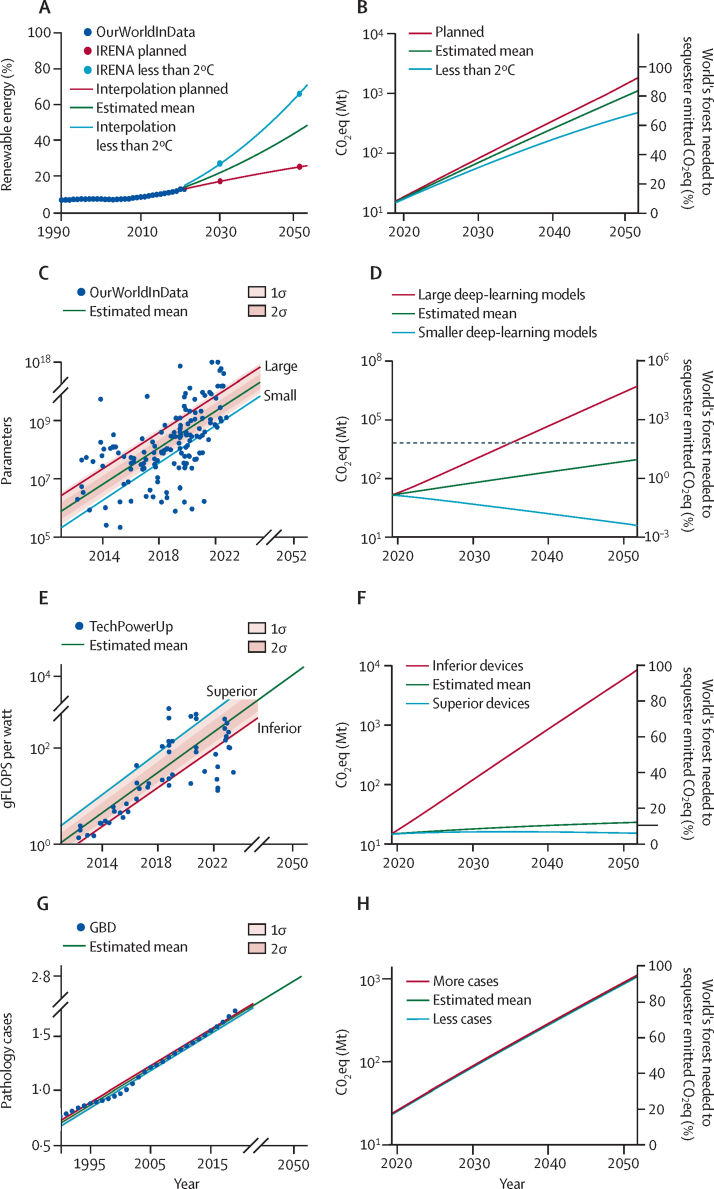

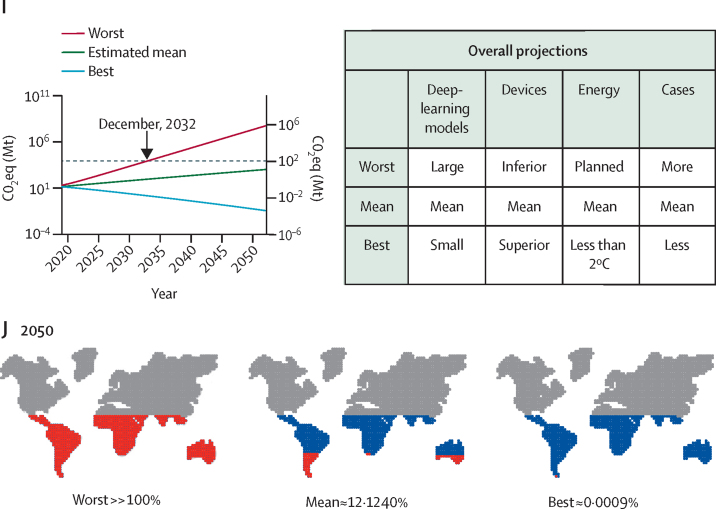
Estimation of global future development of CO_2_ or CO_2_ eq emissions (A) Estimation of renewable-energy development between 2023 and 2052 based on the IRENA global renewables outlook. Past data is from the OurWorldInData database (blue dots). Planned projection (red dots) is based on current energy plans (updated in 2020). Less than 2°C (light blue dots) is an ambitious, potentially realistic projection to keep the increase in global temperature to less than 2°C. Green line is the mean between red and light blue. (B) CO_2_eq emissions for various estimations of renewable-energy development. (C) Projection of development of deep-learning architectures. Lines and shading indicate estimated mean, upper, and lower range in the 95% CI. (D) Corresponding CO_2_eq estimations. Dashed grey line shows the current overall capacity of the world's forest to sequester CO_2_eq. (E) Projection of the development of computational devices. Lines and shading indicate estimated mean, upper, and lower range in the 95% CI. (F) Corresponding CO_2_eq emissions. (G) Projection of development of the number of pathology cases. Lines and dots indicate estimated mean, upper, and lower range in the 95% CI. (H) Corresponding CO_2_eq emissions. (I) Projection of best, worst, and mean CO_2_eq emissions considering the four parameters (ie, development of renewable energy, deep-learning models, computational devices, and number of pathology cases). Grey line shows the current overall capacity of the world's forest to sequester CO_2_eq. (J) World map indicating the current area occupied by forest (blue dots) and the amount of forest required to sequester CO_2_eq emissions (red dots superimposed on top of blue dots) in 2050 in different projections. The multitask scenario for all cases was used as the basis for estimations. σ=statistical sigma (standard deviation). CO_2_=carbon dioxide. CO_2_eq=CO_2_ equivalent. GBD=Global Burden of Diseases, Injuries, and Risk Factors Study. gFLOPS=giga floating point operations per second. IRENA=International Renewable Energy Agency. Mt=megatons.

A theoretical solution for completely environmentally sustainable deep learning in pathology would be a 100% renewable energy source. However, according to Germany's Federal Office for Environment^38^ and the International Renewable Energy Agency reports,^39^ currently, less than 46·2% of electricity generated in Germany and less than 20% worldwide are obtained from renewable sources, with high variability across different countries (figure 5A; appendix p 7). These projections suggest a steady increase in renewable-energy sources. However, these estimates reach only 55% of the planned projection by 2050 and 86% of the less than 2°C projection (figure 5A).^39^ Even in the less than 2°C projection, CO_2_eq emissions from pathology are estimated to continuously increase (figure 5B; appendix p 9). The depth and complexity of deep-learning models are expected to increase in the future (figure 5C) and show the strongest effect on estimated CO_2_eq emissions (figure 5D; appendix p 9).

The efficacy of computational resources is also expected to increase (figure 5E), becoming the second most influential parameter on CO_2_eq emissions after deep-learning model size (figure 5F; appendix p 9). Ranking was based on comparison of results in figure 5 (B, D, F, H). The wider the difference (between the blue and red line) of a parameter in the estimation in 2050, the more influential it was. This projection could potentially be improved by novel computational approaches (eg, quantum of neuromorphic computing); however, there are currently no data to calculate the effects of such technologies. The number of pathology cases could also influence CO_2_eq emissions, but no data on the predicted number of cases are available. Therefore, we used projections of the number of cancer cases from the Global Burden of Disease database.^40^ These data suggest a steady global increase in the number of cases (figure 5G), with some differences between countries (appendix p 7) but little effect on overall CO_2_eq emissions (figure 5H; appendix p 9).

On the basis of these parameters, we projected CO_2_eq emissions for the worst to the best scenarios at the national (appendix p 9) and international levels (figure 5I, J; appendix p 16). The worst scenario was the upper limit range of the 95% CI of CO_2_eq emissions and the best scenario was the lower limit range of CO_2_eq emissions. In the worst situation, by 2032, emissions from the use of deep learning in pathology would reach amounts requiring the whole world's forest to sequester CO_2_eq. In the mean situation, CO_2_eq emissions would still steadily increase and require 12·12% of the world's forest by 2050. However, if sustainability were to be considered, the best situation could result in a steadily decreasing amount of CO_2_eq, indicating artificial intelligence for digital pathology was sustainable (figure 5I, J; appendix p 9).

We focused on deep-learning implementation; however, other steps of the pathology workflow, such as data acquisition (eg, slide digitisation), are relevant. The power use of our Aperio AT2 scanner was 580 W per hour. Assuming a mean scan time of 1 min per slide, the CO_2_eq for scanning all slides generated in our centre in 1 year would result in approximately 917 kg of CO_2_eq emissions.

The total CO_2_eq emissions of preparing this study were 25·51 kg (appendix pp 10–11, 21–22).

## Discussion

Our findings suggest that deep-learning implementation in pathology could lead to considerable CO_2_eq emissions and global-warming potential. These emissions could restrict or even prohibit the wide use of deep learning. Our findings emphasise the importance of considering CO_2_eq emissions when implementing and developing deep learning in pathology, providing encouraging scenarios and potential solutions towards environmentally sustainable deep learning. The substantial variations in CO_2_eq emissions between different deep-learning models show the importance of model selection and size. Because of the similar structure of deep-learning models, these concepts might be of interest not only for pathology, but for medicine in general and beyond.

Theoretically, if energy requirements were fully met by renewable sources, CO_2_eq emissions produced by the computational requirements for deep-learning implementation would become negligible. However, because of the status and projected development of renewable-energy sources,^41^ this scenario seems unrealistic in both the medium and long term, showing the need for future sustainability analyses and considerations.

There are potential solutions to reduce energy consumption while maintaining or even improving the efficiency of deep-learning models.^42^ One approach is the reduction of input data, which are considerably large in pathology. Instead of the entire WSI, omitting areas without tissue substantially reduces CO_2_eq emissions. This process can be done automatically with a dedicated tissue-detection deep-learning model or other computationally less demanding techniques, thereby supporting a fully automated workflow. Further reduction of emissions could be achieved by selecting a minimally required ROI within the tissue. However, when selecting such ROIs, we do not always know what might be minimally required for different tasks.^43^ Furthermore, any contextual information would be lost, for example of the cancer-surrounding tissue. ROIs could be selected manually (eg, by a pathologist selecting only cancer tissue on a WSI, without any computation). However, this process is time-consuming and, because of decreasing numbers of pathologists, especially in Germany,^37^ does not seem to be a viable long-term option. In general, technological developments in medicine lead to increasingly more and larger data than before.^44^ A similar development could be anticipated in pathology (eg, non-destructive tissue imaging).^45^ Thus, in the future, data size might increase, calling for alternative approaches to reduce CO_2_eq emissions, such as more efficient computational hardware or smaller deep-learning model size.

We focused on approaches to reduce CO_2_eq emissions which are directly linked to digital pathology. The reduction of model complexity for deep learning is one important aspect that does not interfere with the automation of the digital-pathology workflow. Instead of current deep-learning models, the selection of computationally less demanding models can substantially influence CO_2_eq emissions. Newer and larger models are generally assumed to outperform older and simpler ones. However, our previous results have shown that simple deep-learning models perform similarly to more advanced deep-learning models in various tasks in pathology.^5^, ^10^, ^31^ In this study, despite having fewer trainable parameters, a relatively simple deep-learning model outperformed a deeper model while reducing CO_2_eq emissions. Moreover, model pruning, also known as model optimisation or compression, is another approach that can reduce CO_2_eq emissions. RCC-classification models that had been pruned by up to 40% could retain their accuracy while generating 20–30% less CO_2_eq emissions than non-pruned models. These findings suggest that model selection during development is an essential aspect of environmentally sustainable deep learning.

Currently, deep-learning development in pathology and medicine is focused on the best potential performance; sustainability is rarely considered. This is similar to the development of deep-learning model architectures, which have become more complex (figure 1D). However, for specific tasks, performance of less complex deep-learning models can be similar to or even slightly better than state-of-the-art models, but with only a fraction of the CO_2_eq emissions.^10^ Particularly complex models can have large amounts of sparsity (ie, the proportion of weights or connections that can be pruned or set to zero, resulting in a sparse neural network with reduced parameters) that do not contribute to the model's performance for a specific task. Pruning can remove such sparsities, resulting in a more computationally efficient model while retaining performance. Furthermore, alternative approaches, such as pathomics, could substantially reduce CO_2_eq emissions while potentially providing more granular, quantitative, and explainable data than deep-learning models.^35^ The trade-off between deep-learning model performance and operational CO_2_eq emissions is particularly challenging in medicine but hard to enumerate and might need regulatory and political decisions to consider these factors further. To date, we are not aware of any approach for such evaluation and decision making. However, we have shown that several approaches can reduce emissions without compromising performance.

Our study has some limitations. The first limitation is the focus on emissions generated by deep-learning implementation (ie, operational CO_2_eq within the lifecycle-assessment framework). The CO_2_eq emissions of the entire digital-pathology workflow, including data generation, preprocessing, and storage and the CO_2_eq emissions associated with production, transport, service, replacement of hardware, construction, maintenance of server rooms and buildings, and socioeconomic attributes were not considered here. Second, the large variety of tissues and diseases in pathology will require specific deep-learning services. In this Article, we only focused on breast and prostate cancer from a pathology institute based in a university hospital due to the available commercial products and supporting scientific literature. Third, we did not consider model training in our calculations, although training can be associated with considerable computational requirements and CO_2_eq emissions. Fourth, deep learning can be continuously improved via increased data (ie, updated) or retrained on data from a specific centre (ie, calibrated). As current regulatory requirements for medical products in Germany do not permit the use of such evolving deep-learning systems, we did not consider training or retraining in the CO_2_eq emissions calculations. Fifth, access to pathology and digitisation services differs between countries. These differences might result in unequal distribution and progress in the implementation of deep learning, which is hard to model. Variations between countries might be also due to differences in health-care systems and availability of data. However, digital pathology and deep learning are an approach to overcome the lack of expert pathologists worldwide, including in low-income and middle-income countries.^46^, ^47^ Finally, as our global and future extrapolations are not based on accurate data, we instead provided a range of scenarios. As several additional factors and aspects influence the use of deep learning in pathology, these extrapolations are only theoretical; they should be a starting point for further, refined calculations. Thus, the generalisability of our findings is low and should be interpreted with caution.

Digitisation, generation of big data, and use of computationally intensive approaches in medicine will probably continue to increase. Therefore, the medical community, policy decision makers, and the public should be aware of the global-warming potential of these developments. Our study could be a basis for discussion of policy considerations in the implementation of deep learning in medicine. Guidelines, incentives, or regulations could be implemented to encourage researchers and industries to test and implement approaches to reduce CO_2_eq emissions (eg, through the use of computationally less demanding deep-learning models, techniques such as model pruning, and alternative approaches such as pathomics or data reduction strategies).^48^

We aimed to provide an environmental-sustainability analysis of the use of deep learning in digital pathology, which should encourage future data collection for more granular calculation models, and extension to other medical areas. We have provided the CO_2_eq-calculation model for the global community, allowing any pathology centre to model its CO_2_eq burden.

## Data sharing

All data used in this study, excluding the WSIs, are publicly available and can be accessed through the sources that are cited as references. The prepared data and code used for analysis and generating the figures are available on GitLab (https://git-ce.rwth-aachen.de/labooratory-ai/sustainability-study). The data directory does not include patient identifiers. The code has been released under an open-source licence and is accessible for everyone to use and modify.

## Declaration of interests

We declare no competing interests.

## References

[1] Lashof DA, Ahuja DR (1990). Relative contributions of greenhouse gas emissions to global warming. Nature.

[2] Rae CL, Farley M, Jeffery KJ, Urai AE (2022). Climate crisis and ecological emergency: why they concern (neuro)scientists, and what we can do. Brain Neurosci Adv.

[3] UN (2023). The 17 goals. https://sdgs.un.org/goals.

[4] Romero Lauro G, Cable W, Lesniak A (2013). Digital pathology consultations—a new era in digital imaging, challenges and practical applications. J Digit Imaging.

[5] Kather JN, Pearson AT, Halama N (2019). Deep learning can predict microsatellite instability directly from histology in gastrointestinal cancer. Nat Med.

[6] Zheng Y, Cassol CA, Jung S (2021). Deep-learning-driven quantification of interstitial fibrosis in digitized kidney biopsies. Am J Pathol.

[7] Bouteldja N, Klinkhammer BM, Schlaich T, Boor P, Merhof D (2022). Improving unsupervised stain-to-stain translation using self-supervision and meta-learning. J Pathol Inform.

[8] Gupta L, Klinkhammer BM, Seikrit C (2022). Large-scale extraction of interpretable features provides new insights into kidney histopathology—a proof-of-concept study. J Pathol Inform.

[9] Büllow RD, Marsh JN, Swamidass SJ, Gaut JP, Boor P (2022). The potential of artificial intelligence-based applications in kidney pathology. Curr Opin Nephrol Hypertens.

[10] Kers J, Bülow RD, Klinkhammer BM (2022). Deep learning-based classification of kidney transplant pathology: a retrospective, multicentre, proof-of-concept study. Lancet Digit Health.

[11] Jabbarpour A, Mahdavi SR, Vafaei Sadr A, Esmaili G, Shiri I, Zaidi H (2022). Unsupervised pseudo CT generation using heterogenous multicentric CT/MR images and CycleGAN: dosimetric assessment for 3D conformal radiotherapy. Comput Biol Med.

[12] Shiri I, Vafaei Sadr A, Amini M (2022). Decentralized distributed multi-institutional PET image segmentation using a federated deep learning framework. Clin Nucl Med.

[13] He J, Baxter SL, Xu J, Xu J, Zhou X, Zhang K (2019). The practical implementation of artificial intelligence technologies in medicine. Nat Med.

[14] Thrall JH, Li X, Li Q (2018). Artificial intelligence and machine learning in radiology: opportunities, challenges, pitfalls, and criteria for success. J Am Coll Radiol.

[15] Zhou Y, Chia MA, Wagner SK (2023). A foundation model for generalizable disease detection from retinal images. Nature.

[16] Becker JU, Mayerich D, Padmanabhan M (2020). Artificial intelligence and machine learning in nephropathology. Kidney Int.

[17] Bülow RD, Dimitrov D, Boor P, Saez-Rodriguez J (2021). How will artificial intelligence and bioinformatics change our understanding of IgA nephropathy in the next decade?. Semin Immunopathol.

[18] Pandey M, Fernandez M, Gentile F (2022). The transformational role of GPU computing and deep learning in drug discovery. Nat Mach Intell.

[19] Kaack LH, Donti PL, Strubell E, Kamiya G, Creutzig F, Rolnick D (2022). Aligning artificial intelligence with climate change mitigation. Nat Clim Chang.

[20] Bankhead P, Loughrey MB, Fernández JA (2017). QuPath: open source software for digital pathology image analysis. Sci Rep.

[21] Henderson P, Hu J, Romoff J, Brunskill E, Jurafsky D, Pineau J (2020). Towards the systematic reporting of the energy and carbon footprints of machine learning. J Mach Learn Res.

[22] China International Capital Corporation Research, China International Capital Corporation Global Institute (2022). Guidebook to carbon neutrality in China: macro and industry trends under new constraints.

[23] Afzal M, Akhtar AM (2013). Factors affecting carbon sequestration in trees. J Agric Res (Lahore).

[24] Harris NL, Gibbs DA, Baccini A (2021). Global maps of twenty-first century forest carbon fluxes. Nat Clim Chang.

[25] OpenAI (2023). GPT-4 technical report. arXiv.

[26] Chowdhery A, Narang S, Devlin J (2022). PaLM: scaling language modeling with pathways. arXiv.

[27] Dehghani M, Djolonga J, Mustafa B (2023). Scaling vision transformers to 22 billion parameters. arXiv.

[28] Tu T, Azizi S, Driess D (2023). Towards generalist biomedical AI. arXiv.

[29] Driess D, Xia F, Sajjadi MSM (2023). PaLM-E: an embodied multimodal language model. arXiv.

[30] Baykal SI, Bulut D, Sahingoz OK (2018). Comparing deep learning performance on BigData by using CPUs and GPUs. https://www.semanticscholar.org/paper/Comparing-deep-learning-performance-on-BigData-by-Baykal-Bulut/4988cc386ee5a0c5e357836a6572bebe20778e58.

[31] Ghaffari Laleh N, Muti HS, Loeffler CML (2022). Benchmarking weakly-supervised deep learning pipelines for whole slide classification in computational pathology. Med Image Anal.

[32] Ghaffari Laleh N, Truhn D, Veldhuizen GP (2022). Adversarial attacks and adversarial robustness in computational pathology. Nat Commun.

[33] Han S, Mao H, Dally WJ (2015). Deep compression: compressing deep neural networks with pruning, trained quantization and huffman coding. arXiv.

[34] Blalock D, Gonzalez Ortiz JJ, Frankle J, Guttag J (2020). What is the state of neural network pruning?. arXiv.

[35] Hölscher DL, Bouteldja N, Joodaki M (2023). Next-generation morphometry for pathomics-data mining in histopathology. Nat Commun.

[36] Destatis (2023). Gross electricity production in Germany. https://www.destatis.de/EN/Themes/Economic-Sectors-Enterprises//Energy/Production/Tables/gross-electricity-production.html.

[37] Märkl B, Füzesi L, Huss R, Bauer S, Schaller T (2021). Number of pathologists in Germany: comparison with European countries, USA, and Canada. Virchows Arch.

[38] Umwelt Bundesamt (2023). Erneuerbare energien in zahlen. https://www.umweltbundesamt.de/themen/klima-energie/erneuerbare-energien/erneuerbare-energien-in-zahlen#uberblick.

[39] International Renewable Energy Agency (2020). Global Renewables Outlook: energy transformation 2050. https://www.irena.org/publications/2020/Apr/Global-Renewables-Outlook-2020.

[40] Institute For Health Metrics and Evaluation, Global Health Data Exchange (2019). Global Burden of Disease Study 2019 (GBD 2019) data resources. https://ghdx.healthdata.org/gbd-2019.

[41] International Renewable Energy Agency (2020). Global renewables outlook: energy transformation 2050. https://www.irena.org/publications/2020/Apr/Global-Renewables-Outlook-2020.

[42] Bartoldson BR, Kailkhura B, Blalock D (2022). Compute-efficient deep learning: algorithmic trends and opportunities. arXiv.

[43] Kosaraju SC, Hao J, Koh HM, Kang M (2020). Deep-Hipo: multi-scale receptive field deep learning for histopathological image analysis. Methods.

[44] (2020). Big hopes for big data. Nat Med.

[45] Walsh CL, Tafforeau P, Wagner WL (2021). Imaging intact human organs with local resolution of cellular structures using hierarchical phase-contrast tomography. Nat Methods.

[46] Robboy SJ, Gross D, Park JY (2020). Reevaluation of the US pathologist workforce size. JAMA Netw Open.

[47] Piya S, Lennerz JK (2023). Sustainable Development Goals applied to digital pathology and artificial intelligence applications in low- to middle-income countries. Front Med (Lausanne).

[48] Grealey J, Lannelongue L, Saw W-Y (2022). The carbon footprint of bioinformatics. Mol Biol Evol.

