# Supplementary material for: Operational greenhouse-gas emissions of deep learning in digital pathology: a modelling study
Source: Lancet Digit Health. 2023 Nov 22;6(1):e58–69. doi: 10.1016/S2589-7500(23)00219-4 (PMC10728828; doi:10.1016/S2589-7500(23)00219-4)
Supplement: Supplementary appendix [file mmc1.pdf]

THE LANCET
Digital Health

Supplementary appendix

This appendix formed part of the original submission and has been peer reviewed. We post it as supplied by the authors.

Supplement to: Vafaei Sadr A, Bülow R, von Stillfried S, et al. Operational greenhouse-gas emissions of deep learning in digital pathology: a modelling study. *Lancet Digit Health* 2023; published online Nov 22. https://doi.org/10.1016/S2589-7500(23)00219-4.

Appendix

Table of contents

Additional Methods 1
Data gathering 1
CO_2eq calculations 1
CO_2eq reduction approaches 2
Results scale and future extrapolations 3
References: 5
Figures 6
Tables 10

Additional Methods

Data gathering

All cooling and supporting overheads are considered using the power usage effectiveness (PUE) coefficient. The PUE describes the energy consumption efficiency in a computer data center and the average is currently 1.55 as reported in 2022[1,2]. The values may be larger (e.g., 3 times) for old centers and lower values for new centers. The experiment impact tracker extracts the power consumption for all devices and tracks the running procedure, estimating the total consumed energy as follows:

$$e_{total} = PUE \sum_{p} (p_{DRAM} e_{DRAM} + p_{CPU} e_{CPU} + p_{GPU} e_{GPU})$$

Here, p is the occupation percentage, as the memory, CPUs, and GPUs may share resources for other procedures, and e is the energy usage for the resource.

CO_2eq calculations

Our CO_2eq calculation pipeline was developed and implemented using Python (V3.9) on Linux servers (Ubuntu 22.04.1) with specific configurations of a Python environment and the following libraries: cucim (version 22.2.0), cudatoolkit (version 11.6.0), cudnn (version 8.2.1.32), experiment-impact-tracker (version 0.1.9), Keras (version 2.11.0), Keras-unit-collection (0.1.13), openslide (version 3.4.1), openslide-python (version 1.1.2), TensorFlow (version 2.11.0), and Python (version 3.9.10). Our local GPU-equipped server at the Institute of Pathology at RWTH Aachen University Hospital served as local hardware for our experiments.

Normalized computational requirements. To generate comparable CO_2eq emissions values, we ran all DL models on the same defined amount of 10^7 tiles for all models. We customized and updated the experiment impact tracker[3] to be compatible with the Python version we used. Based on the DL scenarios, i.e., 1-, 2-, 7-task, and the number of tiles, i.e., "WSI", "tissue" or "ROI", the final CO_2eq can be calculated as follows:

$$CO_2eq = \sum_{task \in scenario} CO_2eq(task)$$

In which $CO_2eq(task)$ is the CO_2eq of a single DL model within the respective scenario and data input. We repeated the experiment for each DL model five times and utilized the mean average values.

CO_2eq reduction approaches

We have trained different models for the classification of renal cell carcinomas, similar to our previous model[4,5]. We used the same 20 DL classification models as described. This enabled us to compare CO_2eq and diagnostic model performance. The models were trained using the default implementation from the libraries for 20 epochs with flip and shift augmentations and used the Adam optimizer with a decaying learning rate. After the models were trained, we calculated the CO_2eq emission for each model in inference.

We collected 265 consecutive RCC cases from the archives of the Institute of Pathology (RWTH Aachen University Hospital), including cases from 01.01.2011 to 31.12.2018 and specifically selecting major RCC subtypes (papillary, clear cell, and chromophobe RCCs). The RCCs were manually annotated using QuPath 0.2.3 and subsequently tessellated to tiles with an edge length of 512 pixels (256 µm; 0.5µm/px). Image tiles containing less than 40% of tissue were excluded using a brightness threshold during tessellation. The final data set consisted of 47 papillary RCCs, 195 clear cell RCCs, and 22 chromophobe RCCs, from which we selected a random set of 4000 images for each class.

We also evaluated the impact of model pruning on the CO_2eq emissions of two of the best-performing RCC-classification DL models. We used sparsity as the metric to measure the degree of pruning, with sparsity defined as the percentage of weights that were pruned from the model. The initial sparsity was set to zero, and we varied the final sparsity between 0.2, 0.3, 0.4, 0.5, 0.6, and 0.7. The final sparsity value indicated the percentage of weights that were pruned after applying the TensorFlow Model Optimization Toolkit (version 0.7.3), specifically the "tfmot.sparsity.keras.PolynomialDecay" function to prune low-magnitude weights from the model.

Results scale and future extrapolations

To extrapolate our results to national and international scales and estimate future outcomes, we used different databases and statistical modeling approaches.

National and International scale. We considered the percentage of renewable sources and the number of cases for this estimation. We used the "OurWorldInData" database[6] to assess the renewable and non-renewable energy sources in Germany and other countries. For countries with no available data, we used the average value for each continent as an approximation (Appendix p16). Our estimation assumed that computing servers are localized in each country. We used the Global Burden of Disease database[7] to obtain cancer incidence data. We assume all cases in different countries have access to digital pathology services for the estimation. Using the data from our center, we used a ratio of 6.7 slides per case and a 7.4:1 ratio of non-cancer to cancer pathology cases to extrapolate the overall number of slides for Germany. Using the results for Germany and the corresponding data for each country, we estimated the CO_2eq emission for each country as follows:

$$CO2eq(c) \;=\; Co2eq(\text{Germany})\frac{n_c}{n_{\text{Germany}}}\frac{1-a_c}{1-a_{\text{Germany}}}$$

Where c is the given country and n_c is the estimated number of cases per country and a_c the percentage of renewable energy sources. The sum of all countries represented the global CO_2eq burden.

Future estimations. We gathered data on four influential factors, i.e., the development of renewable energy sources, the development of DL architectures, the improvement of computational resources, and projections on cancer cases.

To extrapolate the renewable energy developments between 2023-2052, we used the International Renewable Energy Agency (IRENA) global renewables outlook report[8]. According to the report, the planned projection provides a perspective based on the current plans of governments according to the Paris Agreement, updated in 2020. We fitted a bilinear function utilizing the past data from the "OurWorldInData" database[6] to estimate the percent of renewable energy per year (Figures 5a and 5b; Appendix p9).

To predict the DL model development, we used the calculated CO_2eq of the selected DLs (Appendix p10) and extrapolated the number of parameters (DL size) according to the "OurWorldInData" database[9]. We fitted a polynomial to the logarithmic transformation of the data since the increase in DL size and CO_2eq emission is exponential (Figures 5c and 5d). We conducted a thorough evaluation of the goodness of fit with and without employing the logarithmic transformation, utilizing adjusted R2 as the primary metric for comparison. After comparing the adjusted R2, we determined that the logarithmic transformation provided a significant improvement

in the model's fit, with an adjusted R2 of 0.35, compared to the non-transformed fit, which yielded an adjusted R2 of only 0.03. Based on these results, we chose to utilize the logarithmic transformation in our modeling approach resulting in:

$$n_p = e^{0.742\times \text{year-1479}}$$

Where n_p is the number of parameters.

To estimate the development in computational devices, we collected the data from the "TechPowerUp" database on GPUs[10]. Similar to the DL development, we fitted a polynomial on the logarithmic transformation of the data (Figures 5e and 5f):

$$\Gamma = e^{0.208\times \text{year-415.4}}$$

where Γ is GFLOPS per Watt. The choice of logarithmic transformation was based on a comparison between adjusted R2 to evaluate the goodness of fit between not transformed (0.06) and transformed (0.46) fits.

To project the number of pathology cases, we built the estimation based on cancer cases according to the "Global Burden Disease" database[7] and employed a ratio of 6.7 slides per case and a 7.4:1 ratio of pathology cases to estimate all pathology cases. We employed a polynomial function for this estimation:

$$\text{case} = 4.62\times 10^5\,\text{year} - 9.10\times 10^8$$

We utilized adjusted R2 to evaluate the goodness of fit (R2=0.99).

We assumed four hypothetical future scenarios ranging from the worst to the best (Fig 5i).

To ensure the reliability and robustness of our extrapolative statistical modeling, we employed Bootstrapping with 100,000 iterations. This involved creating multiple samples of the corresponding dataset with replacements from the available data and fitting a new statistical model to each sample. The resulting fitted polynomial functions represented the distribution of potential variations in modeling, with the average, upper, and lower range of the 95% confidence interval representing the potential range for extrapolation models.

References:

1. Lee Y-C, Chen K-Y, Yan W-M, Shih Y-C, Chao C-Y. Evaporative cooling method to improve energy management of overhead downward flow-type data center. *Case Studies in Thermal Engineering* 2023: 102998.
2. Mytton D, Ashtine M. Sources of data center energy estimates: A comprehensive review. *Joule* 2022.
3. Henderson P, Hu J, Romoff J, Brunskill E, Jurafsky D, Pineau J. Towards the systematic reporting of the energy and carbon footprints of machine learning. *The Journal of Machine Learning Research* 2020; **21**(1): 10039-81.
4. Kather JN, Heij LR, Grabsch HI, et al. Pan-cancer image-based detection of clinically actionable genetic alterations. *Nature cancer* 2020; **1**(8): 789-99.
5. Laleh NG, Muti HS, Loeffler CML, et al. Benchmarking weakly-supervised deep learning pipelines for whole slide classification in computational pathology. *Medical image analysis* 2022; **79**: 102474.
6. Ritchie H, Roser M, Rosado P. Data, visualizations and writing relating to energy by Our World in Data. 2022. https://ourworldindata.org/energy (accessed Aug 2, 2023).
7. Lopez AD, Murray CC. The global burden of disease, 1990–2020. *Nature medicine* 1998; **4**(11): 1241-3.
8. International Renewable Energy Agency (IRENA). Global Renewables Outlook: Energy transformation 2050. 2020. https://www.irena.org/publications/2020/Apr/Global-Renewables-Outlook-2020 (accessed Aug 2, 2023).
9. Giattino C, Mathieu E, Broden J, Roser M. Key insights, articles, and charts on Artificial Intelligence. 2022. https://ourworldindata.org/artificial-intelligence (accessed Aug 2, 2023).
10. GPU Specs Database of TechPowerUp. 2023. https://www.techpowerup.com/gpu-specs/ (accessed Aug 2, 2023).

Figures

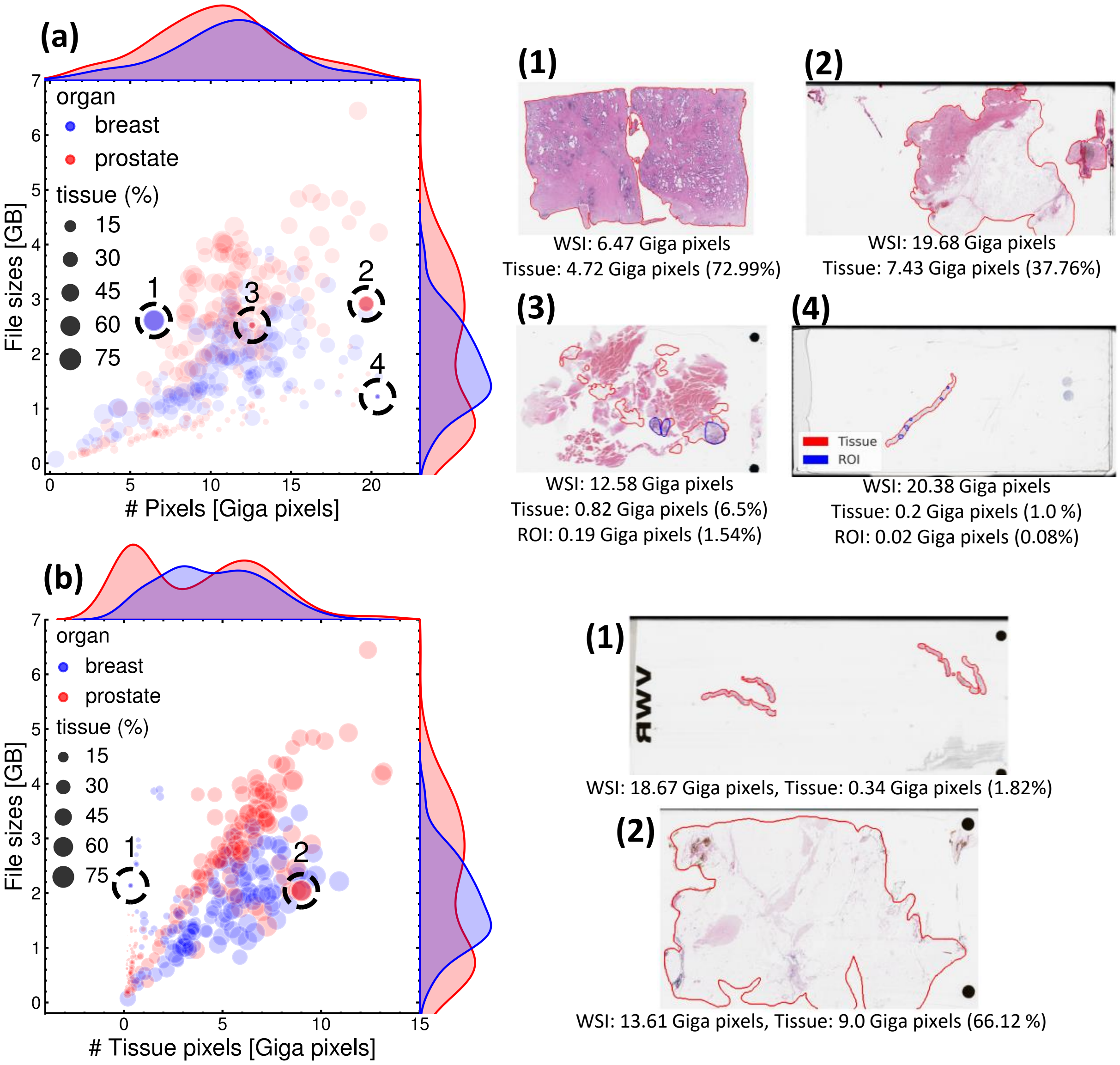

Supplementary Fig 1 *Examples of WSIs. (a) shows the file size distribution versus the number of pixels. Each dot represents one WSI where the area corresponds to the percentage of tissue. Four examples are chosen from different regions in the distribution to get a deeper understanding of the dispersion in the distribution. WSI (1) is an example where the file size is large while the number of pixels is low. WSI (2) shows a case with a similar file size as (1) but significantly more pixels. (3) shows blood contamination and unnecessarily large file size. (4) shows an even smaller file size than (1) and (2) but much more pixels since it includes a large region of background. (b) illustrates the file size versus the number of pixels that include tissue in each WSI. The area of each dot shows the tissue percentage in each WSI. Points indicated as (1) and (2) are examples of slides with different amounts of tissue but similar sizes.*

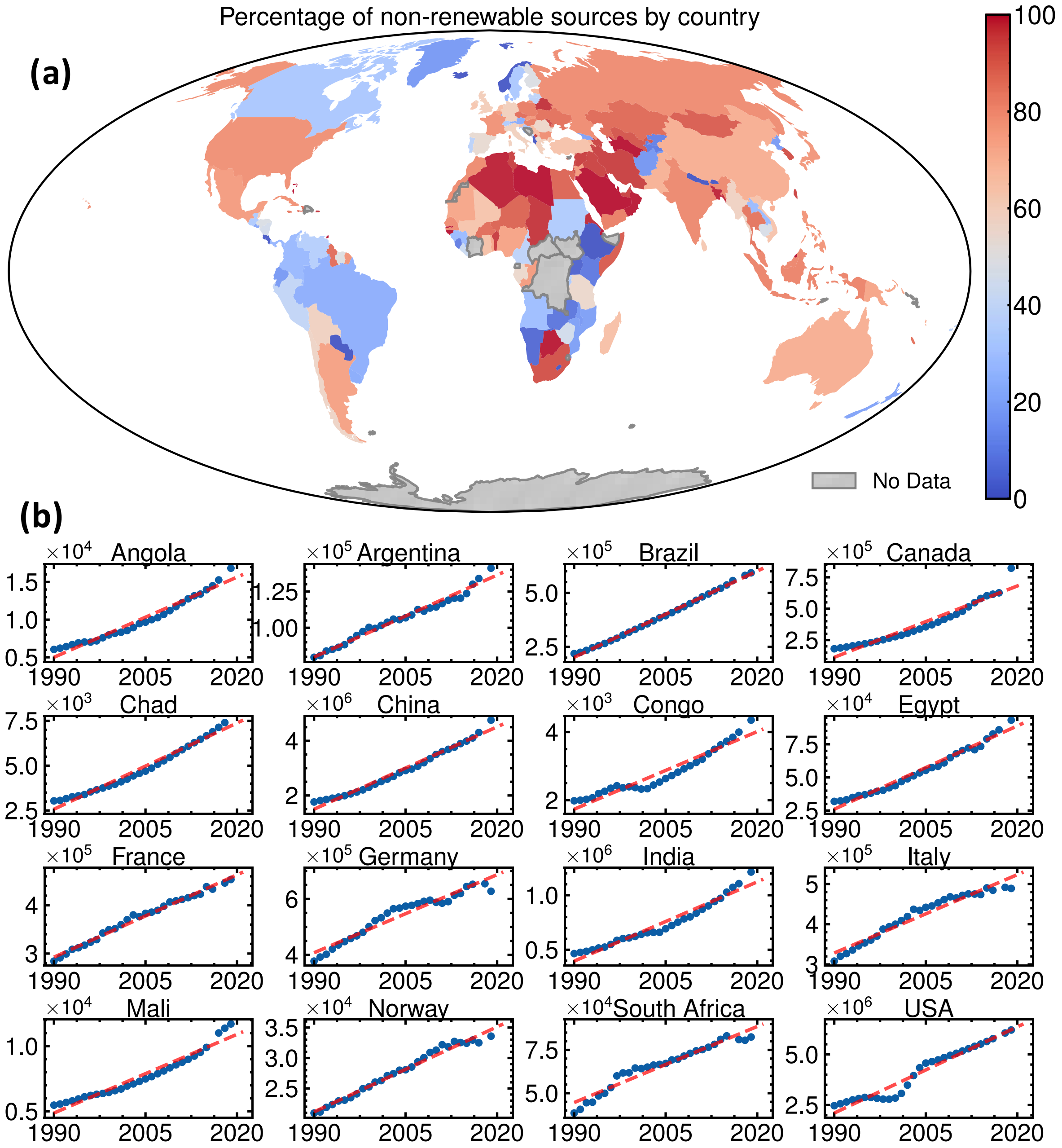

Supplementary Fig 2 *(a) Contribution of renewable and non-renewable sources of energy up to 2022, based on the "OurWorldInData" database. (b) Examples of cancer cases in selected countries based on data from the "Global Burden Disease" database. The figure illustrates the trend of increasing number of cancer cases over time, which can vary depending on the country's population and development.*

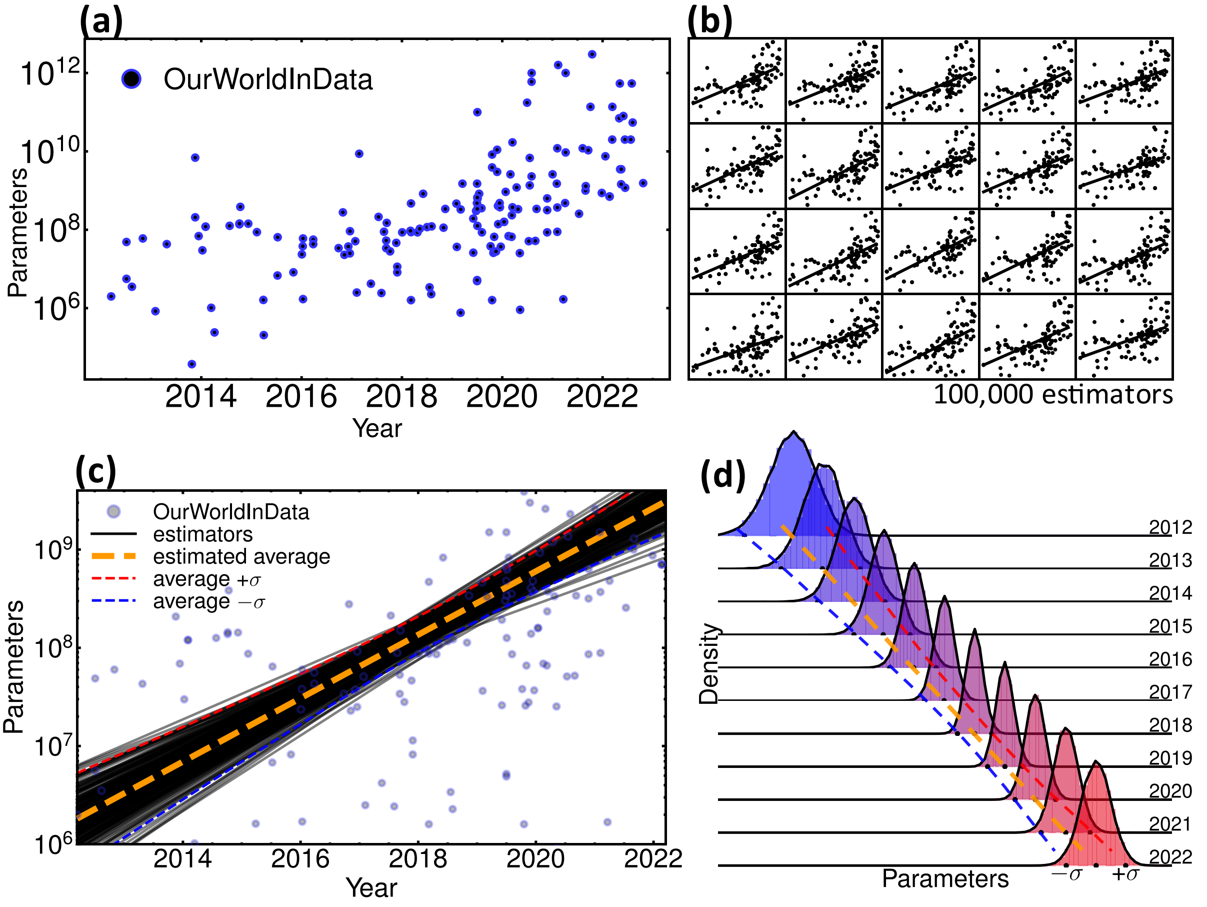

Supplementary Fig 3 *An overview of extrapolative statistical modeling. (a) The number of parameters in deep learning models' data from the "OurWorldInData" database is used as an example. (b) Bootstrapping is used to fit 100,000 linear functions to the logarithmic transformation of the number of parameters. (c) The fitted lines represent a distribution of potential variations in modeling, with the average (orange), upper (red), and lower (blue) range of the 95% confidence interval representing the potential range for extrapolation models. (d) The figure shows the distribution of the fitted models in each year and how the projections are selected.*

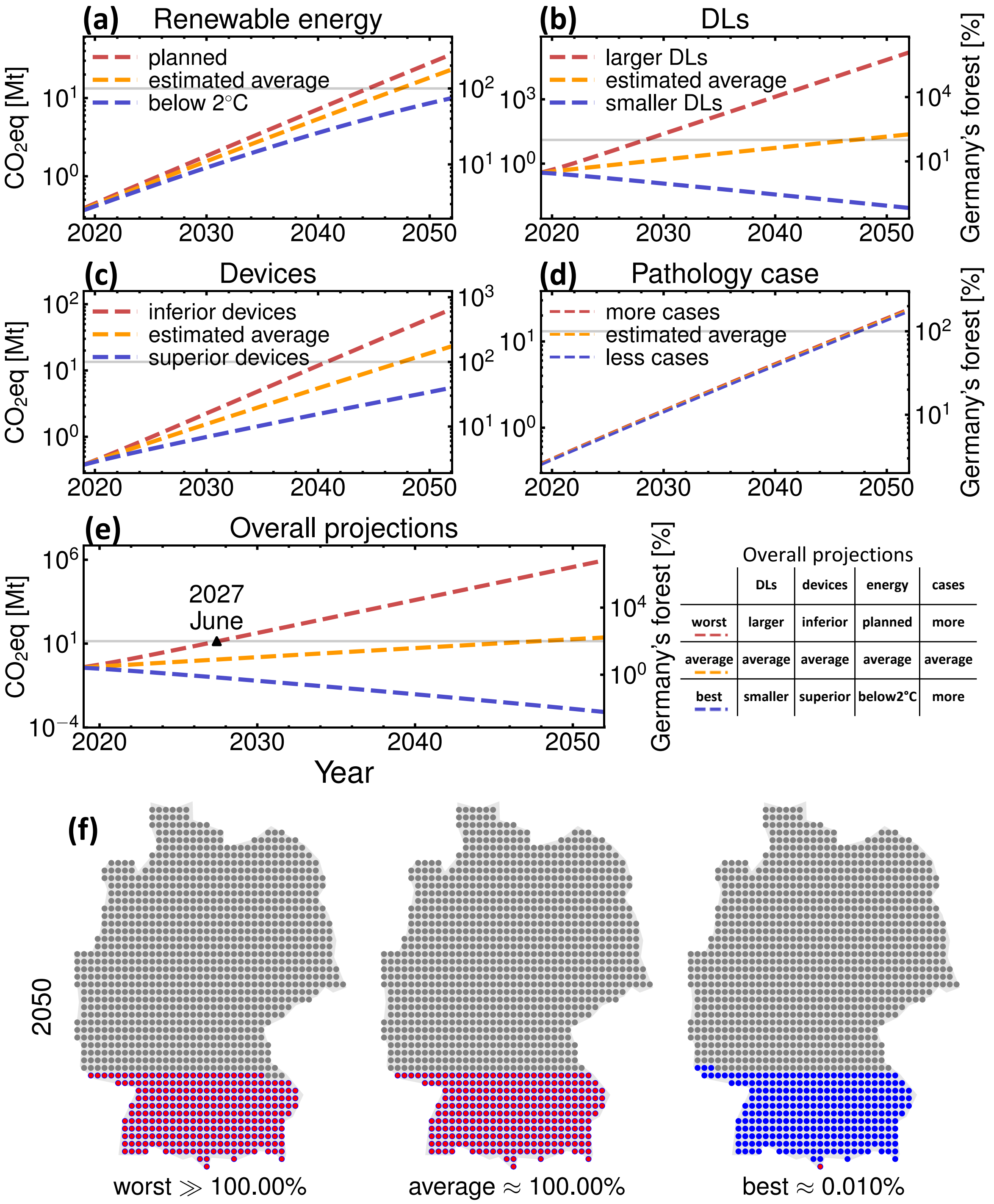

Supplementary Fig 4 *Extrapolation of future potential CO_2 or equivalent (CO_2eq) outcomes considering the multi-task scenario for all cases in Germany. Panel (a) shows the CO_2eq outcomes based on the extrapolation of renewable energy developments between 2023-2052 using the International Renewable Energy Agency (IRENA) global renewables outlook, while panels (b), (c), and (d) show the corresponding outcomes based on the extrapolation of deep learning, computational device, and pathology case developments. Panel (e) shows the best and worst outcomes for all four hypothetical future development projections, associated with the highest and lowest CO_2eq emission in all variables, respectively. The left axis represents the CO_2eq emission, and the right axis indicates how many percent of Germany's forest would be needed to cancel the emitted CO_2eq. The red dots superimposed on blue dots indicate the amount of Germany's forest required, while blue dots represent the remaining forest considering the worst, average, and best projections in 2050 on Germany's map in the panel. The difference in forest area is extrapolated based on historical data on changes of the forest area in Germany for each scenario (f).*

Tables

Supplementary Table 1 Comparison of the number of layers, parameters, and CO_2eq emissions of various machine learning models for classification, segmentation, and multi-task learning tasks.

Task	Model	# layers	# parameters	CO_2eq [kg]
classification	DenseNet121	432	7,334,690	0.26
	DenseNet201	712	18,877,218	0.34
	EfficientNetB0	242	4,420,485	0.24
	EfficientNetB7	818	64,837,241	0.68
	EfficientNetV2B0	260	6,290,226	0.17
	EfficientNetV2B3	388	13,375,264	0.29
	EfficientNetV2L	961	118,117,762	0.69
	EfficientNetV2S	484	20,702,274	0.26
	InceptionResNetV2	785	54,781,378	0.44
	InceptionV3	316	22,394,882	0.25
	MobileNetV3Large	268	3,275,106	0.11
	MobileNetV3Small	234	1,107,282	0.08
	NASNetLarge	1044	86,080,308	0.61
	ResNet152V2	569	58,923,746	0.36
	ResNet50V2	195	24,156,898	0.31
	VGG16	24	14,864,418	0.26
	VGG19	27	20,174,114	0.46
	Xception	137	21,453,578	0.27
	ConvNeXtBase	228	87,568,514	0.95
	ConvNeXtXL	228	348,152,066	2.08
segmentation	Unet 2d	51	31,396,674	1.51
	Vnet 2d	115	18,436,034	0.47
	Attention unet 2d	80	10,161,125	0.75
	Unet plus 2d	76	9,665,352	1.63
	Unet+++ 2d	97	7,891,898	1.71
	R^2unet 2d	156	23,054,466	1.63

	Resunet a 2d	283	52,832,322	3.1
	u2net 2d	609	52,928,660	2.02
	Transunet 2d	152	407,188,802	8.72
	Swin unet 2d	32	9,367,082	1.82
multi-task	PaLM	118	540,350,000,000	77.74

Supplementary Table 2 Comparison of CO_2eq emissions for DL models for breast and prostate tissue cases at RWTH Aachen University Hospital based on a 2019 dataset query. Results for 1-task, 2-task, and 7-task models with small, medium, and large sizes, as well as WSI, tissue, and ROI input scenarios are presented.

**Scenario**	**Input**	**DLs**	**CO_2eq [t] breast**	**CO_2eq [t] prostate**	**CO_2eq [kg/slide] breast**	**CO_2eq [kg/slide] prostate**
1-task	WSI	Small-DL	0.27	0.17	0.01	0.02
		Medium-DL	0.78	0.43	0.04	0.04
		Large-DL	5.05	2.6	0.26	0.26
	Tissue	Small-DL	1.22	0.66	0.06	0.07
		Medium-DL	4.07	2.1	0.21	0.21
		Large-DL	21.62	10.97	1.09	1.08
	ROI	Small-DL	1.2	0.64	0.06	0.06
		Medium-DL	3.98	2.06	0.2	0.2
		Large-DL	20.98	10.7	1.06	1.06
2-task	WSI	Small-DL	1.4	0.75	0.07	0.07
		Medium-DL	4.69	2.41	0.24	0.24
		Large-DL	25.91	13.19	1.31	1.3
	Tissue	Small-DL	1.38	0.73	0.07	0.07
		Medium-DL	4.6	2.34	0.23	0.23
		Large-DL	24.47	12.27	1.24	1.21
	ROI	Small-DL	1.21	0.65	0.06	0.06
		Medium-DL	4.01	2.09	0.2	0.21
		Large-DL	21.15	10.87	1.07	1.07

7-task	WSI	Small-DL	3.34	1.73	0.17	0.17
		Medium-DL	11.44	5.84	0.58	0.58
		Large-DL	66.68	33.88	3.37	3.35
	Tissue	Small-DL	1.64	0.85	0.08	0.08
		Medium-DL	5.52	2.76	0.28	0.27
		Large-DL	30.03	14.82	1.52	1.47
	ROI	Small-DL	1.22	0.67	0.06	0.07
		Medium-DL	4.07	2.14	0.21	0.21
		Large-DL	21.48	11.19	1.09	1.11

Supplementary Table 3 Comparison of the detailed results for extrapolation to all cases for input, DLs, CO_2eq, trees, and forest area for each scenario.

**Scenario**	**Input**	**DLs**	**CO_2eq [t]**	**# trees**	**Forest[m^2]**
1-task	WSI	Small-DL	3.61	165	193
		Medium-DL	9.72	445	519
		Large-DL	60.79	2,788	3,247
	Tissue	Small-DL	15.06	690	804
		Medium-DL	49.04	2,249	2,620
		Large-DL	258.35	11,850	13,801
	ROI	Small-DL	14.7	674	786
		Medium-DL	48.03	2,203	2,566
		Large-DL	251.34	11,529	13,427
2-task	WSI	Small-DL	17.19	788	919
		Medium-DL	56.4	2,587	3,013
		Large-DL	310.12	14,225	16,567
	Tissue	Small-DL	16.84	772	900
		Medium-DL	55.04	2,524	2,940
		Large-DL	290.69	13,334	15,529
	ROI	Small-DL	14.88	682	795

		Medium-DL	48.57	2,227	2,594
		Large-DL	254.35	11,667	13,588
7-task	WSI	Small-DL	40.32	1,849	2,154
		Medium-DL	137.12	6,289	7,325
		Large-DL	797.33	36,574	42,594
	Tissue	Small-DL	19.81	908	1,058
		Medium-DL	65.48	3,003	3,498
		Large-DL	353.95	16,236	18,908
	ROI	Small-DL	15.18	696	811
		Medium-DL	49.51	2,271	2,645
		Large-DL	260.09	11,930	13,894
multi-task	WSI	-	2,795.30	128,225	149,325

Supplementary Table 4 Input, DLs, CO_2eq, and the number of trees and forest area required for each scenario (extrapolation for an estimated 4,643,837 cases in Germany in 2019).

Scenario	Input	DLs	CO_2eq[kt]	# trees	Forest [km^2]
1-task	WSI	Small-DL	0.5	23,883	2
		Medium-DL	1.4	64,261	7
		Large-DL	8.8	401,796	46
	Tissue	Small-DL	2.2	99,538	11
		Medium-DL	7.1	324,176	37
		Large-DL	37.2	1,707,680	198
	ROI	Small-DL	2.1	97,195	11
		Medium-DL	6.9	317,507	36
		Large-DL	36.2	1,661,371	193
2-task	WSI	Small-DL	2.5	113,652	13
		Medium-DL	8.1	372,792	43
		Large-DL	44.7	2,049,883	238

	Tissue	Small-DL	2.4	111,309	12
		Medium-DL	7.9	363,798	42
		Large-DL	41.9	1,921,469	223
	ROI	Small-DL	2.1	98,366	11
		Medium-DL	7	321,021	37
		Large-DL	36.7	1,681,289	195
7-task	WSI	Small-DL	5.8	266,548	31
		Medium-DL	19.8	906,340	105
		Large-DL	114.9	5,270,403	613
	Tissue	Small-DL	2.9	130,921	15
		Medium-DL	9.4	432,837	50
		Large-DL	51	2,339,619	272
	ROI	Small-DL	2.2	100,313	11
		Medium-DL	7.1	327,276	38
		Large-DL	37.5	1,719,177	200
multi-task	WSI	-	402.8	18,477,079	2,151
	Tissue	-	129.3	5,929,325	690
	ROI	-	87.1	3,995,230	465

Supplementary Table 5 Input, DLs, CO_2eq, and the number of trees and forest area required for each scenario (extrapolation for an estimated 173,265,243 cases worldwide in 2019).

Scenario	Input	DLs	CO_2eq[Mt]	# trees	Forest[km ^{2}]
1-task	WSI	Small-DL	0.021	961,110	111
		Medium-DL	0.056	2,585,962	301
		Large-DL	0.352	16,168,715	1,882
	Tissue	Small-DL	0.087	4,005,529	466

		Medium-DL	0.284	13,045,227	1,519
		Large-DL	1.498	68,718,945	8,002
	ROI	Small-DL	0.085	3,911,235	455
		Medium-DL	0.279	12,776,833	1,487
		Large-DL	1.457	66,855,410	7,785
2-task	WSI	Small-DL	0.1	4,573,502	532
		Medium-DL	0.327	15,001,581	1,747
		Large-DL	1.798	82,489,548	9,606
	Tissue	Small-DL	0.098	4,479,209	521
		Medium-DL	0.319	14,639,630	1,704
		Large-DL	1.686	77,322,044	9,004
	ROI	Small-DL	0.086	3,958,382	460
		Medium-DL	0.282	12,918,274	1,504
		Large-DL	1.475	67,656,908	7,879
7-task	WSI	Small-DL	0.234	10,726,184	1,249
		Medium-DL	0.795	36,472,123	4,247
		Large-DL	4.623	212,086,796	24,699
	Tissue	Small-DL	0.115	5,268,430	613
		Medium-DL	0.38	17,417,865	2,028
		Large-DL	2.052	94,148,856	10,964
	ROI	Small-DL	0.088	4,036,714	470
		Medium-DL	0.287	13,169,969	1,533
		Large-DL	1.508	69,181,571	8,056
multi-task	WSI	-	16.209	743,537,832	86,590
	Tissue	-	5.202	238,602,524	27,787
	ROI	-	3.505	160,772,417	18,723

Supplementary Table 6 Comparison of world results by country in 2019: non-renewable energy consumption, pathology cases, CO_2eq emissions, and estimated population.

Country	Non-renewable energy consumption [percent]	Pathology cases	CO_2eq [kt]	Estimated population
Afghanistan	15.66	199,227	4.11	38,041,754
Albania	0	61,925	0.00	2,854,191
Algeria	98.83	325,809	42.42	43,053,054
Angola	28.18	124,580	4.63	31,825,295
Argentina	74.65	1,043,985	102.68	44,938,712
Armenia	68.72	70,570	6.39	2,957,731
Australia	70.87	1,842,444	172.01	25,364,307
Austria	21.96	390,135	11.29	8,877,067
Azerbaijan	94.6	141,892	17.68	10,023,318
Bahamas	100	7,658	1.01	389,482
Bangladesh	98.27	995,537	128.89	163,046,161
Belarus	97.01	301,737	38.57	9,466,856
Belgium	76.32	554,684	55.77	11,484,055
Belize	59.38	3,593	0.28	390,353
Benin	95.83	48,954	6.18	11,801,151
Bhutan	0	4,487	0.00	763,092
Bolivia	58.26	147,557	11.33	11,513,100
Botswana	99.54	23,828	3.13	2,303,697
Brazil	23.23	4,388,241	134.29	211,049,527
Brunei	100	7,094	0.93	433,285
Bulgaria	79.35	327,138	34.20	6,975,761
Burkina Faso	86.86	96,563	11.05	20,321,378
Burundi	33.33	50,325	2.21	11,530,580
Cambodia	47.07	139,468	8.65	16,486,542
Cameroon	37.64	137,854	6.84	25,876,380

Canada	31.83	6,088,127	255.30	37,589,262
Chad	96.77	56,485	7.20	15,946,876
Chile	57.29	445,360	33.62	18,952,038
China	71.09	35,212,772	3,298.06	1,397,715,000
Colombia	26.35	774,791	26.90	50,339,443
Congo	75.87	32,192	3.22	5,380,508
Costa Rica	1.26	99,700	0.17	5,047,561
Croatia	31.42	211,614	8.76	4,067,500
Cuba	78.63	387,781	40.17	11,333,483
Cyprus	84.02	45,801	5.07	1,198,575
Czechia	86.97	541,437	62.04	10,669,709
Denmark	25.95	311,140	10.64	5,818,553
Djibouti	100	7,363	0.97	973,560
Ecuador	16.45	224,323	4.86	17,373,662
Egypt	88.61	690,039	80.56	100,388,073
El Salvador	20.43	81,469	2.19	6,453,553
Eritrea	97.78	35,161	4.53	6,081,196
Estonia	67.95	60,494	5.42	1,326,590
Ethiopia	0.07	396,354	0.04	112,078,730
Fiji	37.17	8,725	0.43	889,953
Finland	48.81	283,454	18.23	5,520,314
France	78.22	3,355,941	345.83	67,059,887
Gabon	55.46	13,591	0.99	2,172,579
Gambia	100	9,453	1.25	2,347,706
Georgia	18.83	99,172	2.46	3,720,382
Germany	59.68	4,643,837	365.13	83,132,799
Ghana	65.14	183,819	15.77	30,417,856
Greece	59.55	545,695	42.81	10,716,322
Greenland	16.67	4,573	0.10	56,225
Guatemala	33.45	156,281	6.89	16,604,026

Guinea	27.34	74,122	2.67	12,771,246
Guinea-Bissau	100	9,229	1.22	1,920,922
Guyana	86.99	8,966	1.03	782,766
Haiti	86.87	104,934	12.01	11,263,077
Honduras	47.95	91,398	5.77	9,746,117
Hungary	83.4	451,869	49.65	9,769,949
Iceland	0.01	12,919	0.00	361,313
India	80.62	8,974,055	953.11	1,366,417,754
Indonesia	81.81	2,295,315	247.39	270,625,568
Iran	95.24	920,919	115.55	82,913,906
Iraq	94.57	318,559	39.69	39,309,783
Ireland	63.15	211,628	17.61	4,941,444
Israel	93.21	248,152	30.47	9,053,300
Italy	58.71	3,618,823	279.92	60,297,396
Jamaica	83.68	54,820	6.04	2,948,279
Japan	77.39	6,566,337	669.48	126,264,931
Jordan	76.46	86,520	8.72	10,101,694
Kazakhstan	87.56	269,828	31.13	18,513,930
Kenya	8.28	209,577	2.29	52,573,973
Kuwait	99.93	32,295	4.25	4,207,083
Kyrgyzstan	10.16	51,818	0.69	6,456,900
Latvia	38.36	75,352	3.81	1,912,789
Lebanon	94.36	123,538	15.36	6,855,713
Lesotho	0	20,860	0.00	2,125,268
Liberia	42.39	19,288	1.08	4,937,374
Libya	99.97	67,329	8.87	6,777,452
Lithuania	37.36	108,165	5.32	2,786,844
Luxembourg	19.42	23,455	0.60	619,896
Madagascar	65.55	107,556	9.29	26,969,307
Malawi	16.2	93,202	1.99	18,628,747

Malaysia	81.41	375,940	40.32	31,949,777
Mali	64.01	86,612	7.30	19,658,031
Mauritania	73.4	19,051	1.84	4,525,696
Mexico	76.06	1,643,356	164.68	127,575,529
Moldova	93.54	83,402	10.28	2,657,637
Mongolia	90.17	53,463	6.35	3,225,167
Montenegro	40.27	22,328	1.18	622,137
Morocco	80.19	321,085	33.92	36,471,769
Mozambique	18.58	133,102	3.26	30,366,036
Myanmar	58.09	492,989	37.73	54,045,420
Namibia	4.46	18,371	0.11	2,494,530
Nepal	0	196,768	0.00	28,608,710
Netherlands	65.71	1,001,560	86.70	17,332,850
New Zealand	19.28	360,871	9.16	4,917,000
Nicaragua	45.36	65,867	3.94	6,545,502
Niger	88.89	66,490	7.79	23,310,715
Nigeria	74.36	779,825	76.39	200,963,599
North Korea	16.82	388,102	8.60	25,666,161
North Macedonia	54.55	70,626	5.08	2,083,459
Norway	0.5	248,541	0.17	5,347,896
Oman	99.56	24,446	3.21	4,974,986
Pakistan	70.26	1,811,776	167.71	216,565,318
Palestine	76.74	36,556	3.70	4,685,306
Panama	25.56	60,096	2.02	4,246,439
Papua New Guinea	73.46	57,108	5.53	8,776,109
Paraguay	0.17	71,470	0.02	7,044,636
Peru	39.39	451,951	23.45	32,510,453
Philippines	77.87	878,971	90.18	108,116,615
Poland	83.35	1,401,957	153.95	37,970,874

Portugal	37.32	508,557	25.01	10,269,417
Puerto Rico	97.2	105,091	13.46	3,193,694
Qatar	99.92	16,640	2.19	2,832,067
Romania	55.88	684,882	50.42	19,356,544
Russia	80.04	4,352,647	458.99	144,373,535
Rwanda	40	69,366	3.66	12,626,950
Saudi Arabia	99.77	244,352	32.12	34,268,528
Senegal	79.86	76,113	8.01	16,296,364
Serbia	67.12	370,049	32.72	6,944,975
Sierra Leone	9.52	35,594	0.45	7,813,215
Slovakia	77.64	224,788	22.99	5,454,073
Slovenia	66.32	104,299	9.11	2,087,946
Somalia	90.24	73,534	8.74	10,192,317
South Africa	92.44	610,201	74.31	58,558,270
South Korea	92.23	1,636,563	198.85	51,709,098
Spain	53.82	2,302,893	163.28	47,076,781
Sri Lanka	62.29	229,771	18.85	21,803,000
Sudan	33.88	187,092	8.35	42,813,238
Suriname	49.5	7,760	0.51	581,363
Sweden	32.79	507,861	21.94	10,285,453
Switzerland	33.94	415,126	18.56	8,574,832
Syria	95.47	109,169	13.73	17,070,135
Taiwan	94.56	833,269	103.81	23,568,378
Tajikistan	8.77	61,180	0.71	9,321,018
Tanzania	55.32	302,460	22.05	58,005,463
Thailand	85.49	1,231,614	138.72	69,625,582
Togo	73.02	36,587	3.52	8,082,366
Trinidad and Tobago	99.89	25,543	3.36	1,394,973
Tunisia	95.22	140,149	17.58	11,694,719

Turkey	64.66	1,349,153	114.92	83,429,615
Turkmenistan	100	47,354	6.24	5,942,089
Uganda	2.95	212,057	0.83	44,269,594
Ukraine	86.04	1,308,462	148.31	44,385,155
United Arab Emirates	95.06	77,815	9.75	9,770,529
United Kingdom	60.22	3,335,823	264.67	66,834,405
United States	79.29	45,922,525	4,797.32	328,239,523
Uruguay	21.93	118,870	3.44	3,461,734
Uzbekistan	91.54	293,070	35.34	33,580,650
Vanuatu	71.43	2,063	0.19	299,882
Venezuela	35.51	533,873	24.98	28,515,829
Vietnam	64.33	1,296,574	109.88	96,462,106
Yemen	82.95	129,846	14.19	29,161,922
Zambia	7.79	97,855	1.00	17,861,030
Zimbabwe	45.02	120,936	7.17	14,645,468

Supplementary Table 7 Accuracy comparison of different models against CO_2eq emission of prediction of 10^7 tiles.

**Model**	**CO_2eq [kg/10^7 tiles]**	**Accuracy**
VGG16	1.05	97.62
DenseNet201	1.35	58.18
ConvNeXtXLarge	8.32	93.4
DenseNet121	1.04	33.81
ConvNeXtBase	3.82	91.39
VGG19	1.85	95.7
Xception	1.08	73.8
MobileNetV3Large	0.42	53.02
MobileNetV3Small	0.34	65.68
ResNet152V2	1.46	56.42

EfficientNetV2S	1.02	43.16
ResNet50V2	1.22	65.98
EfficientNetV2L	2.74	43.09

Supplementary Table 8 Effect of model size on accuracy and CO_2eq: comparison of ConvNeXtXL and VGG16.

**Model**	**Size [%]**	**CO_2eq [kg/10^7 tiles]**	**Accuracy**
ConvNeXtXL	30	6.53	36.13
	40	6.45	36.13
	50	6.58	31.75
	60	6.58	31.34
	70	6.47	55.64
	80	6.48	94.49
	100	8.32	93.4
VGG16	30	0.74	36.24
	40	0.75	56.12
	50	0.8	85.81
	60	0.73	97.34
	70	0.79	98.1
	80	0.78	98.45
	100	1.05	97.62

Supplementary Table 9 Sources of data on energy mixture, DLs, cancer cases, and devices.

	**explanation**	**URL**
Energy mixture[14]	Electricity mixture for all countries	https://ourworldindata.org/electricity-mix
DLs[17]	Data on growth in DL models	https://ourworldindata.org/artificial-intelligence
Cancer cases[15]	Data on cancer incident	https://www.healthdata.org/gbd
Devices[18]	Data on GFLOPs/watt for recent devices	https://www.techpowerup.com/gpu-specs/

Supplementary Table 10 The specification of individual cases used. The cases are from 13 patients where the total number of slides is 363 (140 breast tissue, 223 prostate tissue) including Hematoxylin and Eosin (HE), and Immunohistochemistry (IHC) stains.

**CASE NO.**	**SPECIMEN**	**DIAGNOSIS**	**Gender**	**AGE**	**N SLIDES**	**HE SLIDES**	**IHC SLIDES**
1	breast reduction	mastopathy	f	32	6	6	0
2	prostatectomy	prostate cancer	m	70	62	62	0
2	lymphonodectomy	lymph node	m	70	10	10	0
3	prostate biopsy	prostate cancer	m	70	47	26	21
4	vacuum breast biopsy	breast cancer	f	65	10	6	4
5	vacuum breast biopsy	fibrosis mammae	f	57	14	9	5
6	lump resection	breast cancer	f	80	29	27	2
6	lymph node biopsy	lymph node	f	80	5	3	2
7	lump resection	ductal carcinoma in situ	f	55	19	19	0
8	prostate biopsy	prostate cancer	m	72	26	26	0
9	mastectomy	breast cancer	f	52	28	22	6
10	breast biopsy	fibrosis mammae	f	40	1	1	0
11	prostate transurethral resection	prostate hyperplasia	m	68	8	8	0
12	breast biopsy	ductal carcinoma in situ	f	69	28	21	7
13	lymphonodectomy	lymph node	m	79	10	10	0
13	prostatectomy	prostate cancer	m	79	60	60	0
					**363**	**316**	**47**

Supplementary Table 11 Names of the used DL models per task scenario and parameter size.

	**Small-DL**	**Medium-DL**	**Large-DL**
1-task	1xMobileNetV3Small	1xEfficientNetV2B3	1xConvNeXtXLarge
2-task	1xMobileNetV3Small+<br>1xVnet 2d	1xEfficientNetV2B3+<br>1xUnet-plus 2d	1xConvNeXtXLarge+<br>1xTransunet 2d
7-task	5xMobileNetV3Small+<br>2xVnet 2d	5xEfficientNetV2B3+<br>2xUnet-plus 2d	5xConvNeXtXLarge+<br>2xTransunet 2d
